# Threat Conditioning Prior to Cocaine or Sucrose Exposure Alters Reward-Seeking Behavior in a Sex-Dependent Manner

**DOI:** 10.3390/psychiatryint7020085

**Published:** 2026-04-18

**Authors:** Yobet Perez-Perez, Roberto J. Morales-Silva, Genesis N. Rodriguez-Torres, Rafael Ruiz-Villalobos, Jose C. Rivera-Velez, Edgardo G. Arlequin-Torres, Elaine M. Vera-Torres, Lenin J. Godoy-Muñoz, Serena I. Fazal, Nilenid Rivera-Aviles, Sofia Neira, Marian T. Sepulveda-Orengo

**Affiliations:** 1 Department of Basic Sciences, Ponce Research Institute, Ponce Health Sciences University, Ponce, PR 00716, USA; 2 Department of Psychiatry, University of North Carolina, Chapel Hill, NC 27599, USA

**Keywords:** threat conditioning, acute stress, cocaine, sucrose, sex differences, reinstatement

## Abstract

**Background/Objectives:**

Research has shown a high prevalence of co-occurring trauma-related disorders and cocaine use disorder (CUD). However, there remains a need for preclinical studies to determine how traumatic event exposure influences vulnerability to CUD development and relapse. In this study, we assessed the impact of traumatic event exposure using a threat conditioning (TC) paradigm, which models traumatic event exposure through associative threat learning on cocaine-seeking behavior in adult male and female rats.

**Methods:**

Adult male and female rats were exposed to a single TC session. After TC, the rats underwent cocaine self-administration (SA), extinction training, cue-primed reinstatement, and cocaine-primed reinstatement testing. A parallel cohort was subjected to a sucrose SA cohort to assess whether TC altered non-drug reward seeking in the form of sucrose SA.

**Results:**

In the cocaine cohort, stressed male rats exhibited greater cue- and cocaine-primed reinstatement relative to non-stressed males, whereas no reinstatement differences emerged in female rats. In the sucrose cohort, stressed females displayed increased sucrose pellet delivery during self-administration compared to non-stressed females, but no differences were observed during sucrose reinstatement in either male or female rats.

**Conclusions:**

These findings indicate that trauma exposure prior to cocaine use influences cocaine relapse-related behavior, as well as non-drug reward reinforcement earning, in a sex-specific manner. Overall, these results highlight the value of associative stress models such as TC for studying trauma–addiction comorbidity and the need to investigate the neurobiological mechanisms driving these sex-specific outcomes.

## 1. Introduction

Substance Use Disorder (SUD) and mental illnesses (MIs) represent a significant public health challenge. According to the 2024 National Survey on Drug Use and Health (NSDUH), conducted by the U.S. Substance Abuse and Mental Health Services Administration (SAMHSA), 21.2 million adults experience comorbid SUD and MI [1,2]. MIs include disorders triggered by experiences involving traumas of varying severity, such as trauma- and stressor-related disorders, post-traumatic stress disorder, and unclassified and unspecified trauma disorders, among others [3]. Among SUDs, cocaine use disorder (CUD) remains a major concern, as cocaine continues to be one of the most widely used illicit substances, and no FDA-approved pharmacological treatment currently exists [2]. According to the 2024 NSDUH by SAMHSA, approximately 4.3 million people in the U.S. reported past-year cocaine use, and an estimated 4.3 million individuals met criteria for a past-year CNS stimulant use disorder, a category that includes CUD [1].

Furthermore, studies have identified traumatic event exposure as a contributing factor to the onset of CUD [3–10]. Clinical studies consistently show a high prevalence of trauma-related disorders among individuals with cocaine use disorder, with up to 65% of cocaine-dependent patients having trauma-related disorders and significant traumatic event exposure [9–11]. Specifically, events including childhood trauma and physical, sexual, and emotional abuse are linked to earlier onset, greater severity, and increased likelihood of developing cocaine dependence, with cocaine dependence increasing with the number and types of traumatic experiences [10–13]. Importantly, individuals with trauma-related disorders and CUD exhibit greater resistance to treatment, increased drug craving, and relapse vulnerability compared to those without such a comorbidity [14,15]. Therefore, it is essential to examine the comorbidity of trauma-related disorders and CUD using translational models to understand the molecular, physiological, and neurobiological mechanisms by which traumatic event exposure promotes cocaine seeking and, ultimately, to improve the development of treatment strategies.

Although trauma-related disorders are associated with a higher risk of developing CUD, only a handful of preclinical studies have investigated the effects of stress on cocaine-seeking behavior when the exposure to stress occurs prior to cocaine self-administration (SA) [16–21]. Several preclinical studies have demonstrated that prior exposure to stress at various developmental stages in rats can alter cocaine-seeking behavior [16,17,21]. For instance, early-life exposure to limited bedding and nesting or restraint stress has been shown to increase the acquisition of cocaine self-administration [16,21]. Our own laboratory has also shown that chronic stress exposure in the form of inescapable footshocks prior to cocaine SA induces sex-specific differences in the incubation of cocaine cravings, with stressed females showing greater cue-induced seeking and stressed males showing greater cocaine-induced seeking after abstinence [18]. In contrast, some studies have reported opposing effects, demonstrating that stress exposure during adulthood prior to cocaine administration leads to reductions in cue-primed reinstatement [19] or cocaine self-administration [20]. For example, single prolonged stress (SPS) or modified SPS has been shown to decrease cue-induced reinstatement and cocaine self-administration, respectively. These divergent results suggest that multiple variables, such as the age at which stress or drug exposure occurs, the duration of drug access, and the length of withdrawal, may influence the relationship between stress and drug use. In sum, it is still unclear how trauma-related disorders impact the risk of CUD development, and preclinical models are needed to investigate the physiological and neurobiological connections between the two.

Clinical and preclinical studies have evaluated the psychopathology and neurobiological mechanisms of trauma-related disorders using Pavlovian threat conditioning (TC), also called fear conditioning. TC is a widely used behavioral paradigm for the study of trauma-related disorders, as it allows for the assessment of pathological fear, anxiety-related behaviors, and stress responses through the application of an aversive stimulus in the form of electrical footshocks [22–26]. Clinical research has demonstrated the value of TC in anxiety research, as it facilitates the study of pathological fear through the examination of fear acquisition, extinction, generalization, and avoidance [25]. It has also been used in chronic pain research, showing impaired safety learning and excessive fear generalization in chronic pain patients [26]. Preclinical research has also shown the value of the TC behavioral protocol, as a single exposure to TC triggers PTSD-like phenotypes in rodent models, which appear weeks after the trauma [27].

Despite some advances, the mechanisms by which trauma or threat exposure alters cocaine-seeking behavior remain largely unknown, and this is why our research focuses on the impact of TC prior to cocaine exposure. Rats underwent a single session of TC, followed by a short-access SA protocol, extinction training, and cue-primed and cocaine-primed reinstatement testing. In addition, a separate group of rats underwent sucrose SA instead of cocaine SA to assess the effects of TC on non-drug-seeking behavior in rats of both sexes. Our study showed that TC prior to cocaine exposure induces sex-specific differences in both cocaine- and sucrose-seeking behavior, suggesting that traumatic or threat events before cocaine exposure may influence behavioral vulnerability to CUD in a sex-specific manner.

## 2. Materials and Methods

### 2.1. Animals

Adult (P60) male (250–300 g, *n* = 55) and female (220–280 g, *n* = 75) Sprague Dawley rats were selected prior to surgery. Animals were obtained from the Animal House at Ponce Research Institute. All procedures were approved by the Institutional Animal Care and Use Committee (IACUC) of Ponce Health Sciences University (protocol number: 2202000755C002; approved 15 February 2022) and conducted in accordance with the Guide for the Care and Use of Laboratory Animals of the National Institutes of Health (NIH). Rats were housed individually in standard clear plastic cages and maintained on a 12 h reversed light cycle (0600 to 1800). Rats were housed individually to acclimate them to isolation and to minimize the risk of injury or catheter damage following intrajugular catheterization. Water was available ad libitum, and animals received 18 g of standard rat chow daily (Teklad Global Diets; Madison, WI, USA). Bodyweight was measured daily during self-administration (SA) protocols and reported as percentage change from baseline:

Bodyweight%change=DayX-SADay1/SADay1×100

All behavioral testing was conducted during the dark phase. Animals were handled for 5 days before starting behavioral training.

### 2.2. Operant Training

Rats were subjected to 24 h of food deprivation prior to food training sessions conducted in operant chambers containing two levers. Activation of the designated lever resulted in the delivery of a sucrose pellet (45 mg/pellet, BioServ cat# F0023; Flemington, NJ, USA), whereas pressing the non-designated lever did not yield a reward. Upon reaching a minimum of 100 active lever presses, the animals were removed from the operant chambers and returned to their home cages.

### 2.3. Intrajugular Catheterization

Following the operant training session, animals were sedated with 2–4% isoflurane, and jugular catheterization surgery was performed. A guide cannula (C313G, Plastics One) fitted with silastic tubing (0.025 ID, 0.047 OD Bio-sil) was inserted subcutaneously between the shoulder blades and exited through a 3 mm dermal biopsy hole to facilitate catheter placement. Catheters exiting the skin were stabilized with subdermal surgical mesh (Atrium, Merrimack, NH, USA) and a cannula. The catheter’s other end was implanted 3 cm into the right jugular vein and sutured to the surrounding muscle tissue. After completion of surgery, catheters were flushed every two days with 0.2 mL of 5 mg/mL gentamicin (Cat# 48760, Sigma; Burlington, MA, USA), followed by 0.2 mL of 70 U/mL heparinized saline (Lake Zurich, IL, USA) to maintain patency until cocaine self-administration protocols started. Ketorolac (5 mg/kg) (Lake Forest, IL, USA) was administered intraperitoneally to reduce post-operative pain. A catheter cap was employed to prevent backflow when the rats were not attached to the infusion pumps. Animals were given 5–7 days to recover from surgery before behavioral protocols. Animals were considered recovered and allowed to resume the threat conditioning behavioral protocol once the following criteria were met: a healed catheter incision site without swelling, maintenance of eating and drinking behaviors consistent with pre-operative characteristics, and appropriate weight gain during the post-surgical period [28].

### 2.4. Pavlovian Threat Conditioning

After the post-surgery recovery period, animals were exposed to a single session of Pavlovian threat conditioning (TC). The TC protocol was conducted in a transparent Plexiglas chamber measuring 25.5 × 25.5 × 36 cm (ID#46002, UgoBasile, Gemonio, VA, Italy). The floor consisted of stainless-steel bars designed to deliver electrical shocks. The chamber was situated within a noise-isolating enclosure, and behavioral activity was monitored using a video camera. The session of TC consisted of three habituation tones (30 s, 500 Hz) and seven conditioned tones (30 s, 500 Hz), each paired with a footshock (0.5 s, 0.6 mA) in a two-minute intertrial interval (2 min ITI). Freezing behavior was measured as the time the rats spent immobile during each 30-s tone presentation. A control group of rats received contextual exposure and the same ten tones as the stressed (conditioned) group but without shock presentation. Videos were analyzed using the ANY-Maze Software version 7.61 (Stoelting Co., Wood Dale, IL, USA) for footshock delivery and to track freezing behavior. Videos were also hand-scored by blinded experimenters. Furthermore, darting and hopping behaviors were quantified according to the previously established criteria [29]. Following TC, the rats were placed back in their home cages for five days. Catheters continued to be flushed every two days with 0.2 mL of 5 mg/mL gentamicin, followed by 0.2 mL of 70 U/mL heparinized to maintain patency until cocaine self-administration protocols started.

### 2.5. Self-Administration Behavioral Testing

#### 2.5.1. Cocaine Self-Administration

Five days after TC, intravenous catheters were assessed for patency before starting the short-access cocaine SA. Patency was confirmed by infusing 0.1 mL of 1% propofol (Abbot, Schaumburg, IL, USA) through the catheter, a fast-acting anesthetic that induces immediate muscle tone loss and brief sedation (~5 min). A total of six females were excluded due to patency loss during SA. All sessions were conducted during the dark cycle between 0600 and 1800 h. Cocaine hydrochloride was supplied by the National Institute on Drug Abuse (NIDA, Bethesda, MD, USA) and administered via a syringe pump located outside the training chamber (5 mg/mL in saline, NIDA). Rats were placed in operant conditioning chambers equipped with two retractable levers. Responses on the inactive lever were recorded but not reinforced, while responses on the active lever resulted in cocaine infusion (0.10 mg in 0.020 mL), a 5000 Hz tone, and a 5 s illumination above the lever. The procedure followed a fixed ratio 1 (FR1) schedule with a 20 s time-out after each infusion. Self-administration (SA) sessions were conducted daily for two hours over 12 days. Rats that achieved 10 or more infusions per session progressed to extinction training. Two males and five females were excluded for failing to meet the SA criterion.

#### 2.5.2. Sucrose Self-Administration

A separate group underwent sucrose SA, for which rats were placed in operant conditioning chambers equipped with two retractable levers, as described for cocaine SA. Experimental conditions and the SA criterion were kept the same as in the cocaine SA protocols, with the exception that presses on the active lever resulted in the presentation of a sucrose pellet (45 mg/pellet, TestDiet, Richmond, IL, USA) instead of a cocaine infusion.

#### 2.5.3. Extinction Training

After 24 h of the last SA session, rats underwent 15 days of extinction training, during which presses on both levers were recorded, but these did not result in the presentation of cocaine infusion, light illumination, or tone cue. Extinction sessions were conducted daily for two hours (2 h/day) across 15 consecutive days. Rats progressed to reinstatement testing once they met the extinction criterion, defined as an average of ≤20 active lever presses across the last three extinction sessions. A total of one male and three females of the sucrose group were excluded for not meeting the extinction criterion.

#### 2.5.4. Cue-Primed and Cocaine-Primed Reinstatement

After 24 h from the last extinction session, the animals underwent a two-hour cue-primed reinstatement test in the operant chamber. In the cue-primed test, pressing on the active lever resulted in the presentation of the reward-associated cues (light and tone). To assess reinstatement relative to extinction, we calculated the percentage change in active lever responding during cue-primed reinstatement compared to the extinction baseline, which was defined as the average number of active lever presses across the last three extinction sessions. Percentage change was computed as follows:

Activeleverpress%change=Reinstatementactiveleverpresses-Extinctionbaseline/Extinctionbaseline×100

Animals that failed to reach the reinstatement criterion of >50% above the extinction baseline were excluded. A total of two males and two females from the cocaine group and one female from the sucrose group were excluded for not meeting the cue-primed reinstatement criterion. For females, vaginal smears were collected on day 1 of self-administration and on the day of reinstatement testing to assess whether estrous cycle stage influenced reinstatement responses.

#### 2.5.5. Cocaine-Primed Reinstatement

In the cocaine self-administration (SA) group, extinction training was conducted following cue-primed reinstatement and prior to a subsequent reinstatement protocol. Extinction sessions before cocaine-primed reinstatement lasted for two days or until the mean number of active lever presses during the final two sessions was less than or equal to 25. Upon meeting this criterion, rats underwent a two-hour cocaine-primed reinstatement session, during which cocaine (10 mg/kg, intraperitoneally) was administered before placement in the operant chamber. Lever pressing was recorded but did not elicit presentation of drug-associated cues, such as light or tone. One male rat from the cocaine group was excluded for failing to meet the extinction criterion before cocaine-primed reinstatement. For female rats, vaginal smears were collected on the first day of self-administration and on the day of cocaine-primed reinstatement testing to assess potential effects of the estrous cycle on reinstatement. After reinstatement testing, rats completed threat extinction, extinction retrieval, and open field tests, as described below.

### 2.6. Threat Extinction and Extinction Retrieval

The behavioral protocols were conducted as follows: threat extinction was conducted after two days in the home cage following reinstatement testing, and extinction retrieval was conducted 24 h after threat extinction. For threat extinction, animals were subjected to a 14-tone (500 Hz, 2 min ITI) extinction protocol. On the following day, they were exposed to a 2-tone (500 Hz, 2 min ITI) extinction retrieval protocol. All videos were analyzed using the ANY-Maze Software (Stoelting Co., Wood Dale, IL, USA) for tracking freezing behavior. Videos were also hand-scored by blinded experimenters.

### 2.7. Open Field Test

Following extinction retrieval, the animals underwent an open field protocol to measure locomotor activity and anxiety-like behavior. The open field protocol consisted of a 1 h baseline recording, followed immediately by a second 1 h recording after a 10 mg/kg, i.p. cocaine injection, for a total 2 h open field session. The total distance traveled, as well as the time spent in the center and lateral zones, were analyzed to assess differences in locomotor activity and anxiety-like behavior between groups. In addition, cocaine-induced locomotion increase was assessed using the final 30 min of the baseline session and the first 30 min post-cocaine injection. For the sucrose groups, only a 1 h open field test was conducted. Rats were placed in an open field chamber (45 cm × 45 cm, 40 cm high walls) under red light conditions, and their behavior was video-tracked using the ANY-Maze Software (Stoelting Co., Wood Dale, IL, USA). After the behavioral studies, animals were anesthetized with an intraperitoneal injection of pentobarbital (65 mg/mL) and then decapitated.

### 2.8. Statistical Analysis

All data were analyzed using Prism GraphPad version 10.6.1 (GraphPad Software, Inc., La Jolla, CA, USA). Lever presses, cocaine infusions, sucrose pellet deliveries, bodyweight percentage change, threat conditioning, threat extinction, threat conditioning retrieval, extinction retrieval, and distance traveled in the open field test of cocaine or sucrose SA were analyzed using ANOVA to assess multiple comparisons between treatment groups and sessions, followed by post hoc uncorrected Fisher’s LSD tests to evaluate significant effects (Tables S1–S3). A two-way ANOVA was conducted to compare cue-primed, cocaine-primed reinstatement, and cocaine-induced locomotion between groups, with Tukey’s multiple comparison tests used to assess significant effects (Table S2). Center time and lateral time data were analyzed using *t*-tests. The normality of each dataset was assessed prior to analysis. Normally distributed data were analyzed using unpaired *t*-tests, whereas non-normally distributed data were analyzed using Mann–Whitney tests. Data are reported as standard deviation (SD), with statistical significance defined as *p* < 0.05.

## 3. Results

### 3.1. Threat Conditioning in Male and Female Rats Prior to Self-Administration

**Figure 1.**
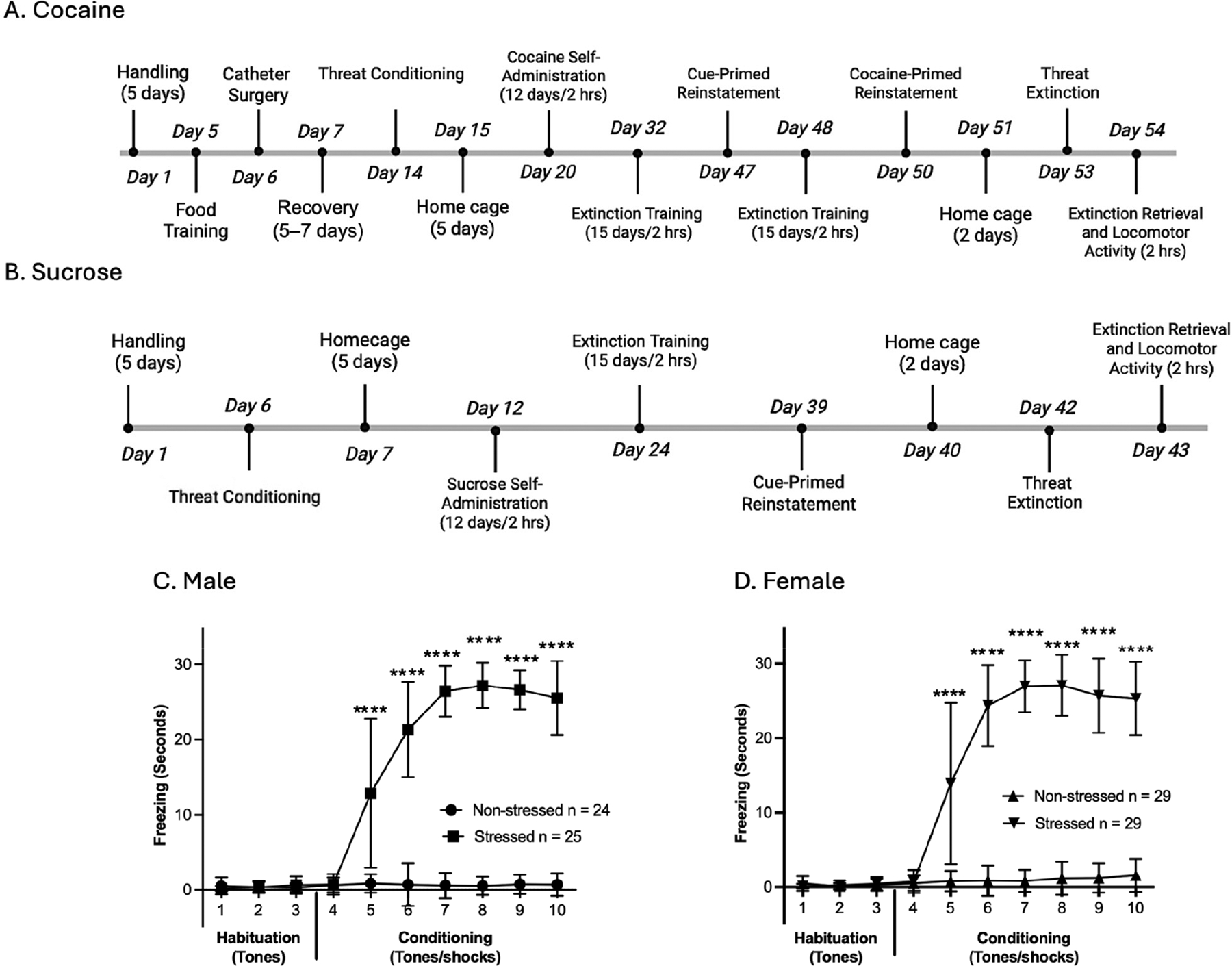
Stressed rats of both sexes show higher freezing compared to their non-stressed counterparts. (**A**) Cocaine group experimental timeline. (**B**) Sucrose group experimental timeline. (**C**) Freezing during tone presentation in stressed males that received auditory threat conditioning and non-stressed males that received tone and context exposure. (**D**) Freezing during tone presentation in stressed females that received auditory threat conditioning and non-stressed females that received tone and context exposure. **** Indicates statistical significance shown by post hoc test (*p* < 0.0001).

To determine how traumatic event exposure influenced freezing behavior prior to cocaine or sucrose SA, rats were subjected to TC (Figure 1) (experimental timelines, Figure 1A,B). A two-way repeated measures ANOVA was conducted for TC analysis. There were significant main effects of time, treatment, and time × treatment interaction in both males [F(2.160, 101.5) = 201.3, *p* < 0.0001; F(1, 47) = 1011, *p* < 0.0001; F(2.160, 101.5) = 195.2, *p* < 0.0001; (Figure 1C)] and females [F(2.528, 141.6) = 222.8, *p* < 0.0001; F(1, 56) = 1013, *p* < 0.0001; F(2.528, 141.6) = 197.7, *p* < 0.0001; (Figure 1D)]. Post hoc comparisons confirmed that both stressed males and females had significantly higher freezing during tone–shock pairings compared to their non-stressed counterparts (tone-alone trials) on tones 5 through 10 during TC (* = *p* < 0.05, uncorrected Fisher’s LSD test; Figure 1C,D). In addition to freezing, a minority of animals demonstrated active conditioned responses. Darting was observed in 12% (*n* = 3) of stressed males and 3% (*n* = 1) of stressed females, whereas hopping occurred in 4% (*n* = 1) of stressed males and 3% (*n* = 1) of stressed females (Figure S1). Taken together, results showed that stressed rats of both sexes froze more than their non-stressed counterparts, confirming the successful acquisition of the conditioned threat training. Following the TC, rats were returned to their home cages for five days to consolidate threat memory.

### 3.2. Self-Administration of Cocaine and Sucrose Groups

**Figure 2.**
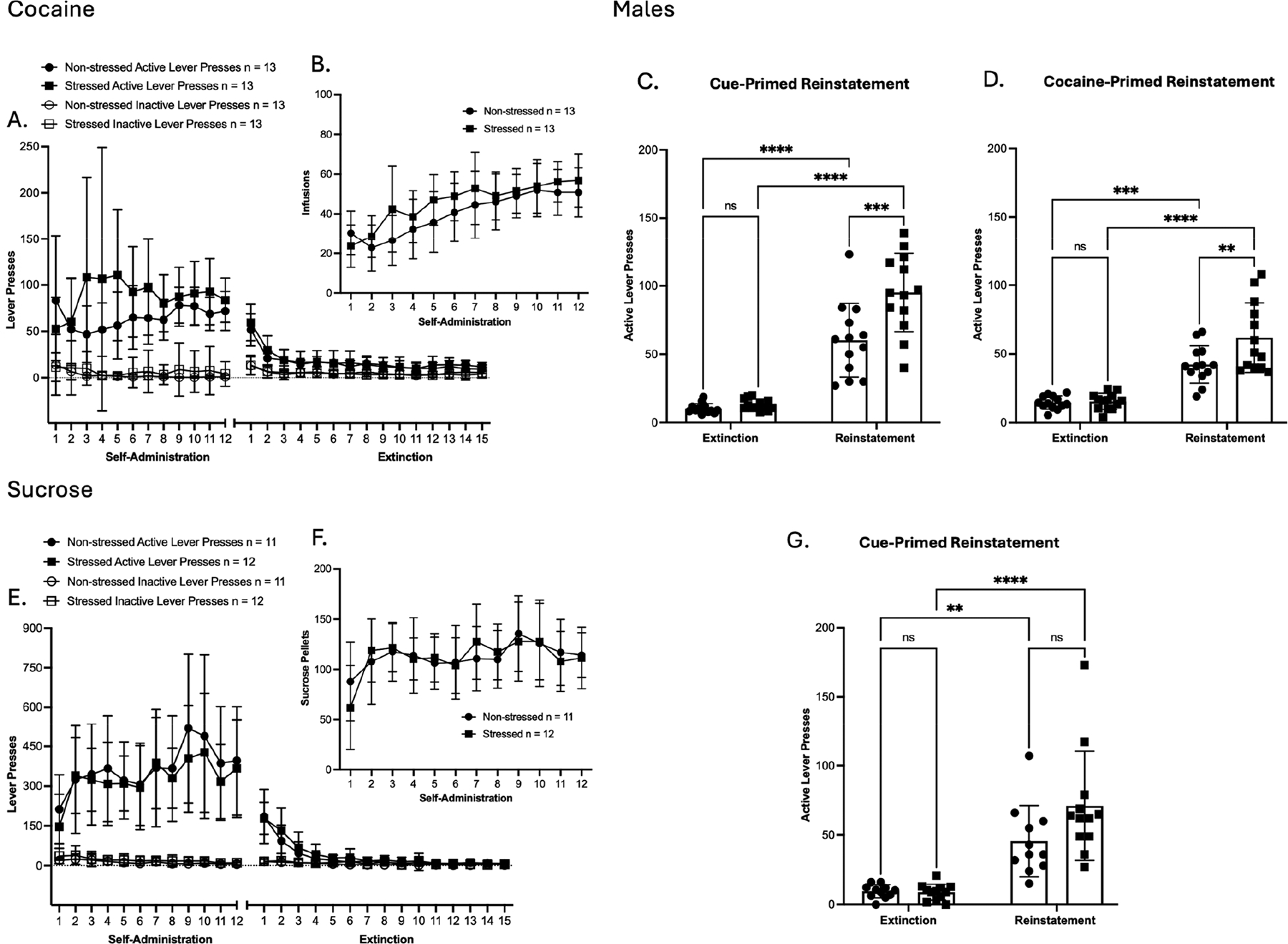
Threat conditioning administered prior to self-administration increased both cue-primed and cocaine-primed reinstatement in the male cocaine group but did not affect the male sucrose group. (**A**) Lever presses during cocaine self-administration and extinction training in male rats across groups. (**B**) Cocaine infusions in male rats across groups. (**C**) Reinstatement in males following presentation of cocaine-paired cues, measured by active lever presses (right) and average lever presses during the last three days of extinction training for each group (left). (**D**) Reinstatement in males following cocaine injection, measured by active lever presses (right) and average lever presses during the last two days of extinction training for each group (left). (**E**) Lever presses during sucrose self-administration and extinction training in male rats across groups. (**F**) Sucrose pellet deliveries in male rats across groups. (**G**) Reinstatement in males following presentation of sucrose-paired cues, measured by active lever presses (right) and average lever presses during the last three days of extinction training for each group (left). **, ***, **** Indicate statistical significance as determined by post hoc test (*p* < 0.01, 0.001, 0.0001, respectively). ns Indicates the non-statistical difference.

**Figure 3.**
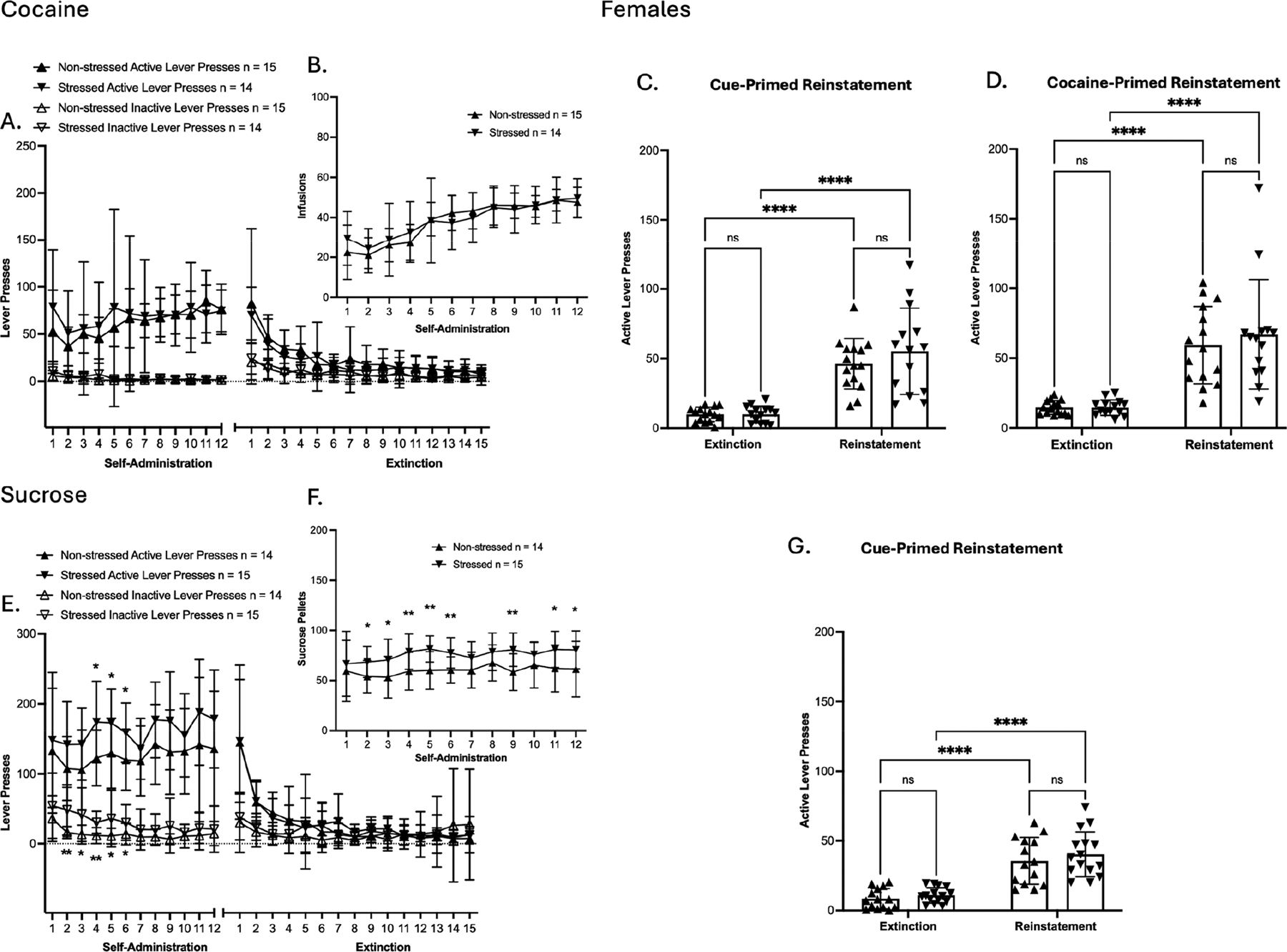
Threat conditioning prior to self-administration increased sucrose self-administration in the female sucrose group but did not affect the female cocaine group. (**A**) Lever presses during cocaine self-administration and extinction training for female rats across groups. (**B**) Cocaine infusions received by female rats across groups. (**C**) Reinstatement in response to cocaine-paired cues, measured by active lever presses (right) and average lever presses during the last three days of extinction training for each group (left). (**D**) Reinstatement following cocaine injection, measured by active lever presses (right) and average lever presses during the last two days of extinction training for each group (left). (**E**) Lever presses during sucrose self-administration and extinction training for female rats across groups. (**F**) Sucrose pellet deliveries for female rats across groups. (**G**) Reinstatement in response to sucrose-paired cues, measured by active lever presses (right) and average lever presses during the last three days of extinction training for each group (left). *, **, **** Indicate statistical significance shown by post hoc test (*p* < 0.05, 0.01, 0.0001, respectively). ns Indicates the non-statistical difference.

Following threat memory consolidation, rats underwent cocaine or sucrose SA (Figures 2 and 3). A two-way ANOVA indicates no significant main effects in self-administration (SA) active or inactive lever presses among cocaine-exposed male rats (Figure 2A). For reward delivery, a significant main effect of time was observed in cocaine infusion [F(4.168, 100.0) = 28.65, *p* < 0.0001; (Figure 2B)]. During SA extinction training, active and inactive lever presses showed a significant main effect of time [F(5.200, 124.8) = 49.82, *p* < 0.0001; F(4.874, 117.0) = 7.790, *p* < 0.0001, respectively (Figure 2A)]. Bodyweight % change across SA (Figure S2) showed a significant main effect of time [F(2.472, 59.32) = 367.3, *p* < 0.0001; (Figure S2A)]. Taken together, these results show that prior TC did not alter cocaine SA or extinction in males, as lever pressing, reward delivery, and bodyweight % change were similar between stressed and non-stressed male groups. Importantly, significant main effects of time were observed across measures, which show successful SA acquisition, as well as the successful extinction of SA behavior among male rats. Twenty-four hours after the final extinction session, the animals underwent a cue-primed reinstatement test. A two-way ANOVA showed a significant main effect of time in active lever presses compared to the average of the last three days of extinction [F(1, 48) = 143.7, *p* < 0.0001; (Figure 2C)]. In addition, within cue-primed reinstatement groups, significant main effects of treatment and time × treatment interaction were observed [F(1, 48) = 11.78, *p* = 0.0012; F(1, 48) = 8.555, *p* = 0.0052; (Figure 2C)]. Post hoc test confirmed that stressed males displayed greater cue-induced cocaine seeking than non-stressed males (*p* = 0.0002, Tukey’s multiple comparison test). Clearly, TC selectively enhanced cue-induced seeking in cocaine males, with stressed males displaying greater reinstatement than non-stressed males. Afterwards, a cocaine-primed reinstatement test was conducted after additional extinction sessions. Results showed a significant main effect of time in active lever presses compared to the average of the last two days of post-cue extinction sessions [F(1, 48) = 80.90, *p* < 0.0001; (Figure 2D)]. In addition, within cocaine-primed reinstatement groups, significant main effects of treatment and time × treatment interaction were observed [F(1, 48) = 6.076, *p* = 0.0173; F(1, 48) = 5.231, *p* = 0.0266 (Figure 2D)]. Post hoc test confirmed that stressed males displayed greater cocaine-induced seeking than non-stressed males (*p* = 0.0081, Tukey’s multiple comparison test). As in the cue-primed test, stressed males also had greater cocaine-primed reinstatement, indicating that TC facilitated cocaine seeking following drug priming.

Similar to cocaine SA, a two-way ANOVA indicates no significant main effects were observed in self-administration (SA) active or inactive lever presses among sucrose-exposed male rats (Figure 2E). However, SA active and inactive lever presses showed a significant main effect of time [F(4.606, 96.73) = 8.230, *p* = <0.0001; [F(3.747, 78.69) = 5.901, *p* = 0.0004, respectively (Figure 2E)]. In reward delivery, a significant main effect of time was observed in sucrose pellet delivery [F(4.096, 86.02) = 7.981, *p* < 0.0001; (Figure 2F)]. During SA extinction training, active and inactive lever presses showed a significant main effect of time [F(2.323, 48.79) = 58.16, *p* < 0.0001; F(3.595, 75.50) = 5.615, *p* = 0.0008, respectively (Figure 2E)]. Bodyweight % change across SA (Figure S2) showed a significant main effect of time [F(2.063, 43.33) = 357.5, *p* < 0.0001; (Figure S2B)]. Taken together, these results show that prior TC did not alter sucrose SA or extinction in males, as lever pressing, reward delivery, and bodyweight % change were similar between stressed and non-stressed male groups. Importantly, significant main effects of time were observed across measures, which show successful SA acquisition, as well as successful extinction of SA behavior among male rats. Twenty-four hours after the final extinction session, the animals underwent a cue-primed reinstatement test. A significant main effect of time was observed in active lever presses compared to the average of the last three days of extinction [F(1, 42) = 48.22, *p* < 0.0001; (Figure 2G)]. However, within the cue-primed reinstatement groups, no differences were observed in active lever presses between non-stressed and stressed males. Overall, non-drug reinstatement remained unaffected.

In females of the cocaine group, no significant effects were observed in SA active lever presses (Figure 3A). In SA inactive lever presses, a significant main effect of time was observed [F(4.602, 124.2) = 8.076, *p* < 0.0001; (Figure 3A)]. In reward delivery, a significant main effect of time was observed in cocaine infusion [F(3.225, 87.06) = 33.34, *p* < 0.0001; (Figure 3B)]. In SA extinction training, active and inactive lever presses showed a significant main effect of time [F(2.679, 72.32) = 19.57, *p* < 0.0001; [F(4.654, 125.6) = 9.184, *p* < 0.0001, respectively (Figure 3A)]. Bodyweight % change across SA showed significant main effects of time and time × treatment interaction [F(3.879, 104.7) = 58.89, *p* < 0.0001; F(3.879, 104.7) = 3.343, *p* = 0.0137; (Figure S2C)]. Taken together, these results show that prior TC did not alter cocaine SA, as lever pressing, infusions, and bodyweight % change were similar between stressed and non-stressed female groups. Importantly, significant main effects of time across measures reflect successful SA acquisition, as well as the successful extinction of SA behavior among females. In cue-primed reinstatement, a significant main effect of time was observed in active lever presses compared to the average of the last three days of extinction [F(1, 54) = 72.63, *p* < 0.0001; (Figure 3C)]. In cocaine-primed reinstatement, a significant main effect of time was observed in active lever presses compared to the average of the last two days of post-cue extinction sessions [F(1, 54) = 58.48, *p* < 0.0001; (Figure 3D)]. However, within the reinstatement groups, no differences were observed in active lever presses between non-stressed and stressed females in either cueor cocaine-primed reinstatement. In addition, the estrous cycle stage, which was assessed by vaginal smears collected on day 1 of SA and on the day of reinstatement testing, did not show any influence on reinstatement responses. In general, these results show that prior TC exposure did not influence cocaine SA or reinstatement behavior in the cocaine group of females under either the cue- or drug-priming condition.

In females of the sucrose group, SA active lever presses showed a significant main effect of treatment [F(1, 27) = 5.220, *p* = 0.0304; (Figure 3E)]. Post hoc comparisons confirmed that stressed females had significantly higher active lever presses during sucrose SA compared to their non-stressed counterparts on days 4–6 (* = *p* < 0.05, uncorrected Fisher’s LSD test; Figure 3E). In SA inactive lever presses, significant main effects of time and treatment were observed [F(4.338, 117.1) = 4.962, *p* = 0.0007; F(1, 27) = 10.18, *p* = 0.0036; (Figure 3E)]. Post hoc comparisons confirmed that stressed females had significantly higher inactive lever presses during sucrose SA compared to their non-stressed counterparts on days 2–6 (* = *p* < 0.05, uncorrected Fisher’s LSD test; Figure 3E). In reward delivery, a significant main effect of the treatment was observed in sucrose pellet delivery [F(1, 27) = 9.783, *p* = 0.0042; (Figure 3F)]. Post hoc comparisons confirmed that stressed females had significantly higher sucrose pellet delivery during sucrose SA compared to their non-stressed counterparts on days 2–6, 9, 11, and 12 (* = *p* < 0.05, uncorrected Fisher’s LSD test; Figure 3F). In SA extinction training, active lever presses showed a significant main effect of time [F(2.403, 64.87) = 33.97, *p* < 0.0001; (Figure 3E)], and no significant main effects were observed in inactive lever presses. Bodyweight % change across SA showed a significant main effect of time [F(5.589, 150.9) = 268.3, *p* < 0.0001; (Figure S2D)]. Taken together, these results show that prior TC altered sucrose SA, as stressed females had higher active lever presses and earned significantly more sucrose pellets than non-stressed females. Importantly, significant main effects of time across measures reflect successful SA acquisition, as well as the successful extinction of SA behavior among females. In cue-primed reinstatement, a significant main effect of time was observed in active lever presses compared to the average of the last three days of extinction [F(1, 54) = 74.21, *p* < 0.0001; (Figure 3G)]. However, within the cue-primed reinstatement groups, no differences were observed in active lever presses between non-stressed and stressed females. In addition, the estrous cycle stage, which was assessed by vaginal smears collected on day 1 of SA and on the day of reinstatement testing, did not show any influence on reinstatement responses. In general, these results show that prior TC exposure caused an increase in sucrose SA but did not influence reinstatement behavior in the sucrose group of females.

In summary, males showed no differences between stressed and non-stressed groups across SA, indicating that prior TC did not alter cocaine or sucrose SA. However, during reinstatement, TC selectively increased cocaine seeking, as stressed males exhibited significantly higher cue-primed and cocaine-primed reinstatement than controls, while sucrose reinstatement remained unaffected. In females, prior TC did not alter self-administration in the cocaine group nor reinstatement in either the cocaine or sucrose groups. However, stressed females had higher active lever presses and sucrose pellet delivery than non-stressed females, indicating enhanced sucrose SA. Overall, TC selectively potentiated cocaine reinstatement in males and non-drug reward SA in females.

### 3.3. Threat Extinction and Extinction Retrieval of Cocaine or Sucrose Self-Administration Groups

**Figure 4.**
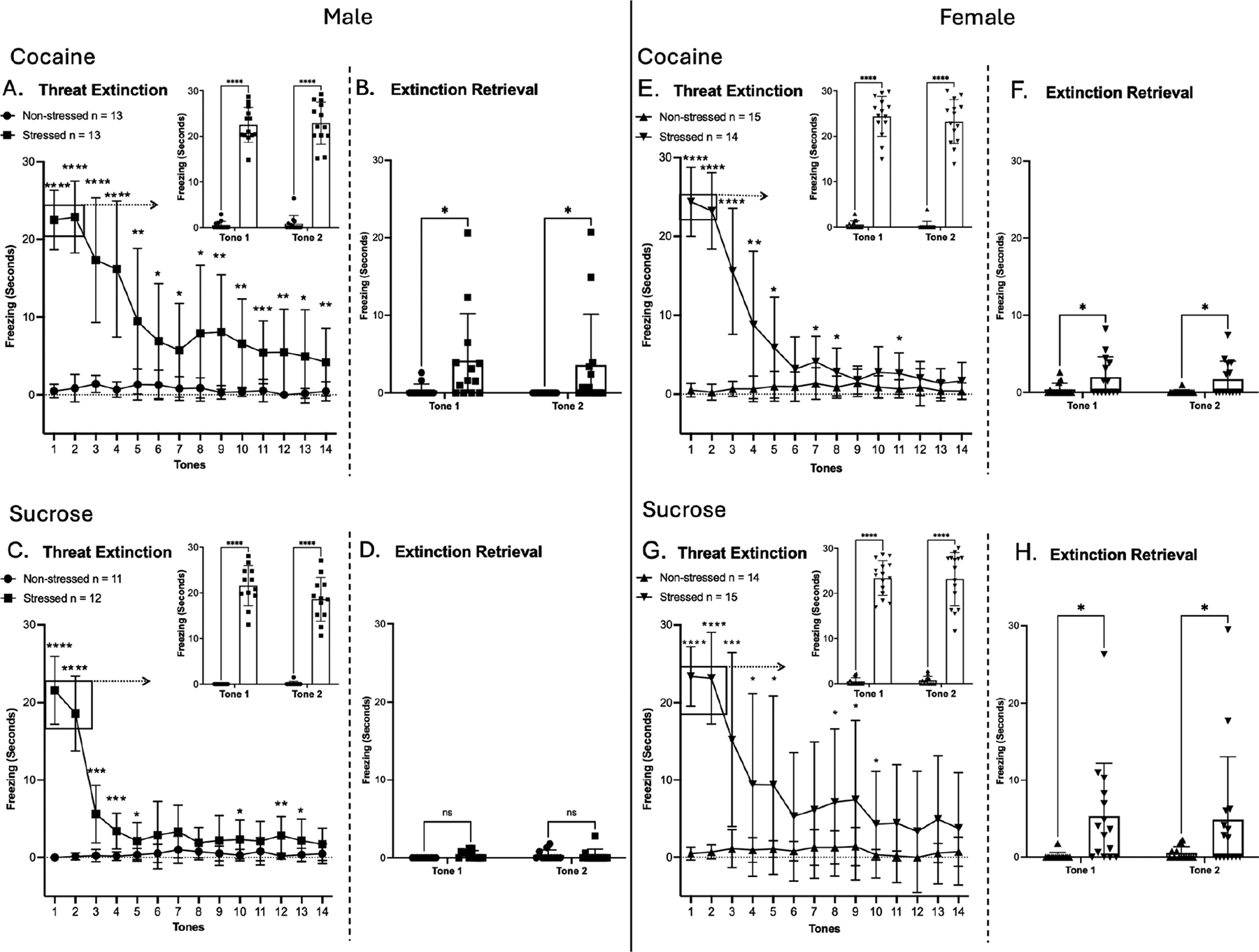
Freezing during threat extinction, threat conditioning retrieval, and extinction retrieval was higher in both stressed male and stressed female rats in the cocaine group, as well as in stressed females in the sucrose group, compared to their non-stressed counterparts. However, in stressed males from the sucrose group, freezing was higher on threat extinction and threat conditioning retrieval, with no differences observed during extinction retrieval compared to their non-stressed counterparts. (**A**) Threat extinction (day 53) of the male cocaine group. The first two tones of the threat extinction of males in the cocaine group represent threat conditioning retrieval. (**B**) Extinction retrieval (day 54) of the male cocaine group. (**C**) Threat extinction (day 42) of males in the sucrose group. The first two tones of the threat extinction of the male sucrose group represent threat conditioning retrieval. (**D**) Extinction retrieval (day 43) of males in the sucrose group. (**E**) Threat extinction (day 53) of the female cocaine group. The first two tones of the threat extinction of the female cocaine group represent the threat conditioning retrieval. (**F**) Extinction retrieval (day 54) of the female cocaine group. (**G**) Threat extinction (day 42) of the female sucrose group. The first two tones of the threat extinction of the female sucrose group represent threat conditioning retrieval. (**H**) Extinction retrieval (day 43) of the female sucrose group. *, **, ***, **** Indicate statistical significance shown by post hoc test (*p* < 0.05, 0.01, 0.001, 0.0001, respectively). ns Indicates the non-statistical difference.

Following reinstatement testing, rats remained in their home cages for two days before undergoing a fourteen-tone threat extinction test (Figure 4). A two-way repeated measures ANOVA was conducted for freezing behavior during threat extinction, TC retrieval, and extinction retrieval sessions. Consistent with earlier findings, sex-specific differences were observed, and thus, the results for males and females are presented individually below.

In males of the cocaine SA group, significant main effects of time, treatment, and time × treatment interaction were observed on threat extinction (day 53; experimental timeline, Figure 1A) [F(4.792, 115.0) = 21.15, *p* < 0.0001; F(1, 24) = 58.54, *p* < 0.0001; F(4.792, 115.0) = 19.57, *p* < 0.0001; (Figure 4A)]. Post hoc comparisons confirmed that stressed males had significantly higher freezing during threat extinction compared to their non-stressed counterparts across all 14 tone-alone trials (* = *p* < 0.05, uncorrected Fisher’s LSD test; Figure 4A). In TC retrieval (the first two tones of threat extinction), a significant main effect was observed for treatment [F(1, 24) = 610.4, *p* < 0.0001; (Figure 4A, insert)]. At extinction retrieval, a significant main effect of treatment was observed (day 54; experimental timeline, Figure 1A) [F(1, 24) = 4.938, *p* = 0.0360; (Figure 4B)].

In males of the sucrose SA group, significant main effects of time, treatment, and time × treatment interaction were observed on threat extinction (day 42; experimental timeline, Figure 1B) [F(5.378, 112.9) = 47.69, *p* < 0.0001; F(1, 21) = 93.37, *p* < 0.0001; F(5.378, 112.9) = 52.49, *p* < 0.0001; (Figure 4C)]. Post hoc comparisons confirmed that stressed males had significantly higher freezing during threat extinction compared to their non-stressed counterparts during tones 1–5, 10, 12, and 13 (* = *p* < 0.05, uncorrected Fisher’s LSD test; Figure 4C). In TC retrieval, a significant main effect was observed for treatment [F(1, 21) = 401.0, *p* < 0.0001; (Figure 4C, insert)]. At extinction retrieval, no significant main effects were observed (day 42, Figure 4D; experimental timeline, Figure 1B).

Taken together, these results indicate that stressed males exhibited greater freezing during threat extinction and TC retrieval compared to their non-stressed counterparts, confirming the persistence of conditioned threat memory following cocaine and sucrose SA. Also, although stressed males in the cocaine group showed a progressive reduction in freezing across tone presentations, extinction learning was attenuated when compared to the sucrose group. This interpretation is supported by the fact that stressed males had higher freezing than non-stressed males at extinction retrieval in the cocaine group only, suggesting that cocaine experience contributes to a more persistent threat-conditioned memory when paired with prior TC exposure.

In females of the cocaine SA group, significant main effects of time, treatment, and time × treatment interaction were observed on threat extinction [F(4.167, 112.5) = 45.72, *p* < 0.0001; F(1, 27) = 109.6, *p* < 0.0001; F(4.167, 112.5) = 49.70, *p* < 0.0001; (Figure 4E)]. Post hoc comparisons confirmed that stressed females had significantly higher freezing during threat extinction compared to their non-stressed counterparts during tones 1–5, 7, 8, and 11 (* = *p* < 0.05, uncorrected Fisher’s LSD test; Figure 4E). In TC retrieval, there was a significant main effect of treatment [F(1, 27) = 485.1, *p* < 0.0001; (Figure 4E, insert)]. At extinction retrieval, females showed a significant main effect of treatment [F(1, 27) = 8.581, *p* = 0.0068; (Figure 4F)].

In females of the sucrose SA group, significant main effects of time, treatment, and time × treatment interaction were observed on threat extinction [F(4.539, 122.6) = 15.70, *p* < 0.0001; F(1, 27) = 23.32, *p* < 0.0001; F(4.539, 122.6) = 15.30, *p* < 0.0001; (Figure 4G)]. Post hoc comparisons indicated that stressed females had significantly higher freezing during threat extinction compared to their non-stressed counterparts during tones 1–5 and 8–10 (* = *p* < 0.05, uncorrected Fisher’s LSD test; Figure 4G). In TC retrieval, there was a significant main effect of treatment (F(1, 27) = 366.2, *p* < 0.0001; (Figure 4G, insert)]. At extinction retrieval, females showed a significant main effect of treatment [F(1, 27) = 5.987, *p* = 0.0212; (Figure 4H)].

Taken together, these results show that stressed females exhibited greater freezing across threat extinction and TC retrieval compared to their non-stressed counterparts, confirming the persistence of conditioned threat memory after cocaine and sucrose SA. However, in contrast to males, stressed females maintained elevated freezing in extinction retrieval in both the cocaine and sucrose groups, suggesting a more robust influence of prior TC independent of reward type.

### 3.4. Open Field Test of Cocaine and Sucrose Self-Administration Groups

**Figure 5.**
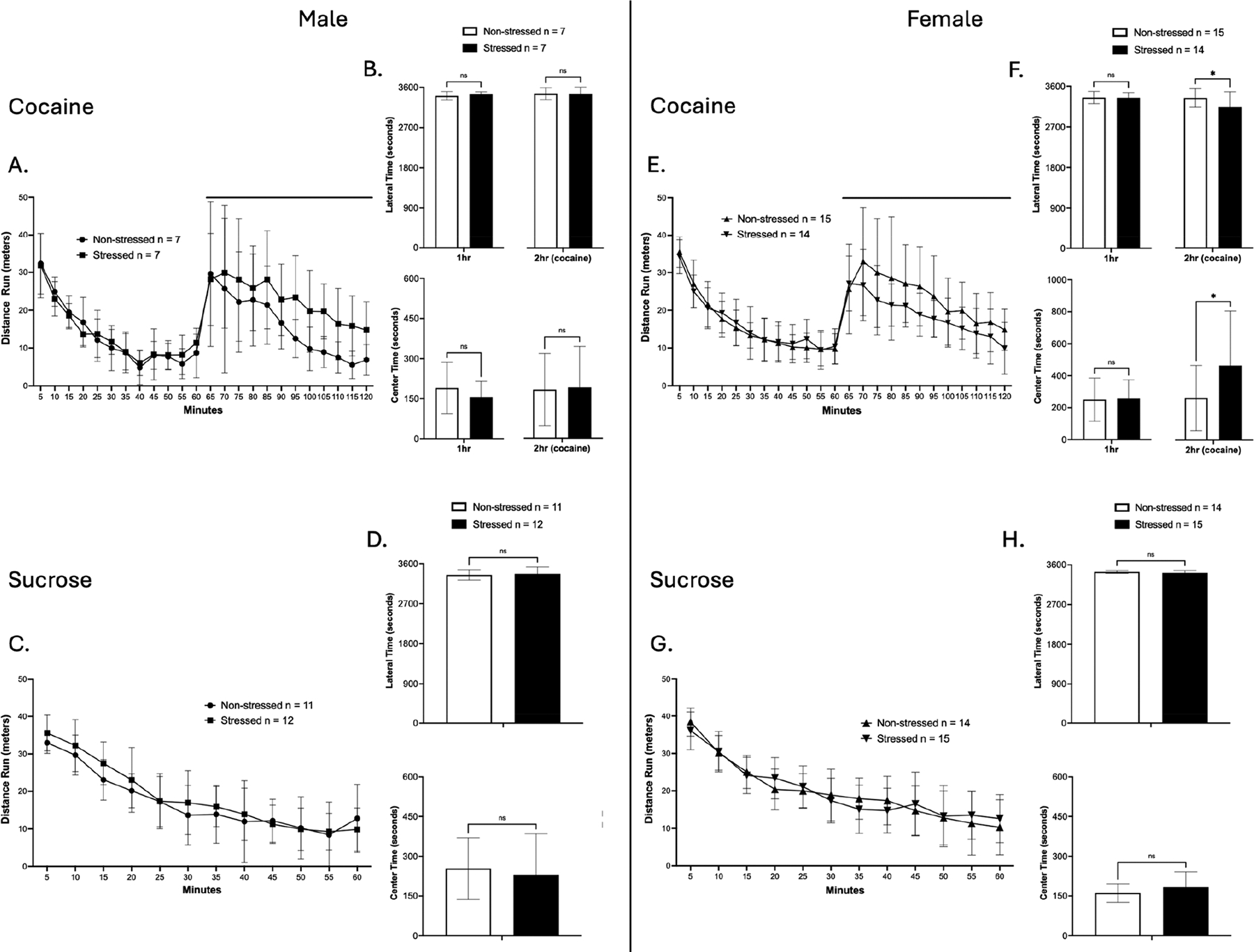
Stressed males and females exposed to cocaine did not differ in locomotor activity compared to non-stressed counterparts. However, stressed females exhibited a cocaine-induced reduction in anxiety-like behavior relative to controls, an effect not observed in males. In the sucrose groups, no differences in locomotor activity or anxiety-like behavior were detected between groups of either sex. (**A**) Male cocaine group, open field total distance divided into 5 min bins across baseline (first 60 min) and after cocaine (10 mg/kg/mL) IP injection (last 60 min), represented by the black bar. (**B**) Male cocaine group, open field lateral time and center time across baseline (1 h) and after cocaine (10 mg/kg/mL) IP injection (2 h). (**C**) Male sucrose group, open field total distance divided into 5 min bins. (**D**) Male sucrose group, open field lateral time and center time. (**E**) Female cocaine group, open field total distance divided into 5 min bins across baseline (first 60 min) and after cocaine (10 mg/kg/mL) IP injection (last 60 min), represented by the black bar. (**F**) Female cocaine group, open field lateral time and center time across baseline (1 h) and after cocaine (10 mg/kg/mL) IP injection (2 h). (**G**) Female sucrose group, open field total distance divided into 5 min bins. (**H**) Female sucrose group, open field lateral time and center time. * Indicates statistical significance shown by post hoc test (distance) or *t*-test analysis (lateral and center time) (*p* < 0.05). ns Indicates the non-statistical difference.

Following extinction retrieval, locomotor activity and anxiety-like behavior were assessed in cocaine and sucrose SA groups (Figure 5). Rats were recorded for one hour at baseline, followed by a second one-hour session under the effects of cocaine injection for the cocaine SA groups or a single one-hour session for the sucrose SA groups. Locomotor activity, measured as distance traveled, was analyzed using a two-way repeated measures ANOVA. Anxiety-like behavior, measured as time spent in the lateral versus center zones, was analyzed using *t*-tests.

In males of the cocaine SA group, a significant main effect of time was observed on locomotor activity in both the first hour [F(4.124, 49.49) = 47.87, *p* < 0.0001; (Figure 5A)] and the second hour [F(2.538, 30.45) = 11.78, *p* < 0.0001; (Figure 5A)]. When assessing cocaine-induced hyperlocomotion, comparing baseline vs post-cocaine injection, a significant main effect of time was observed [F(1, 24) = 25.19, *p* < 0.0001; (Figure S4)]. Post hoc test confirmed that both non-stressed and stressed males displayed cocaine-induced hyperlocomotion (*p* = 0.0168, *p* = 0.0040; Tukey’s multiple comparison test). However, no differences were observed between non-stressed and stressed males in cocaine-induced hyperlocomotion. In addition, there were no differences in anxiety-like behavior measurements (Figure 5B).

In males of the sucrose SA group, a significant main effect of time was observed on locomotor activity [F(5.254, 110.3) = 68.68, *p* < 0.0001; (Figure 5C)]. There were no differences in anxiety-like behavior measurements (Figure 5D). Taken together, results show that prior TC did not alter either locomotor activity or anxiety-like behavior in males.

Similar to males, females of the cocaine SA group had a significant main effect of time that was observed on locomotor activity in both the first hour [F(6.463, 174.5) = 136.8, *p* < 0.0001; (Figure 5E)] and the second hour [F(3.735, 100.9) = 22.24, *p* < 0.0001; (Figure 5E)]. When assessing cocaine-induced hyperlocomotion, there was a significant main effect of time observed [F(1, 54) = 65.15, *p* < 0.0001; (Figure S4)]. Post hoc test confirmed that both non-stressed and stressed females displayed cocaine-induced hyperlocomotion (*p* < 0.0001, *p* = 0.0002; Tukey’s multiple comparison test). However, no differences were observed between non-stressed and stressed females in cocaine-induced hyperlocomotion. In addition, a significant difference was found in anxiety-like behavior analysis on the second hour of the session (Mann–Whitney U = 57, *p* = 0.0367; Figure 5F), showing that stressed females had lower anxiety-like behavior when compared to non-stressed females.

In females of the sucrose SA group, a significant main effect of time was observed on locomotor activity [F(5.822, 157.2) = 78.77, *p* < 0.0001; (Figure 5G)]. In addition, no differences were observed in anxiety-like behavior (Figure 5H). Taken together, results show that prior TC did not alter overall locomotor activity in females. However, stressed females had less anxiety-like behavior under cocaine exposure. In summary, TC did not influence locomotion or anxiety-like behavior in either sex, but it selectively enhanced behavioral responses under cocaine exposure, decreasing anxiety-like behavior in females.

### 3.5. Threat Conditioning Prior to Self-Administration—Sex Comparisons

**Figure 6.**
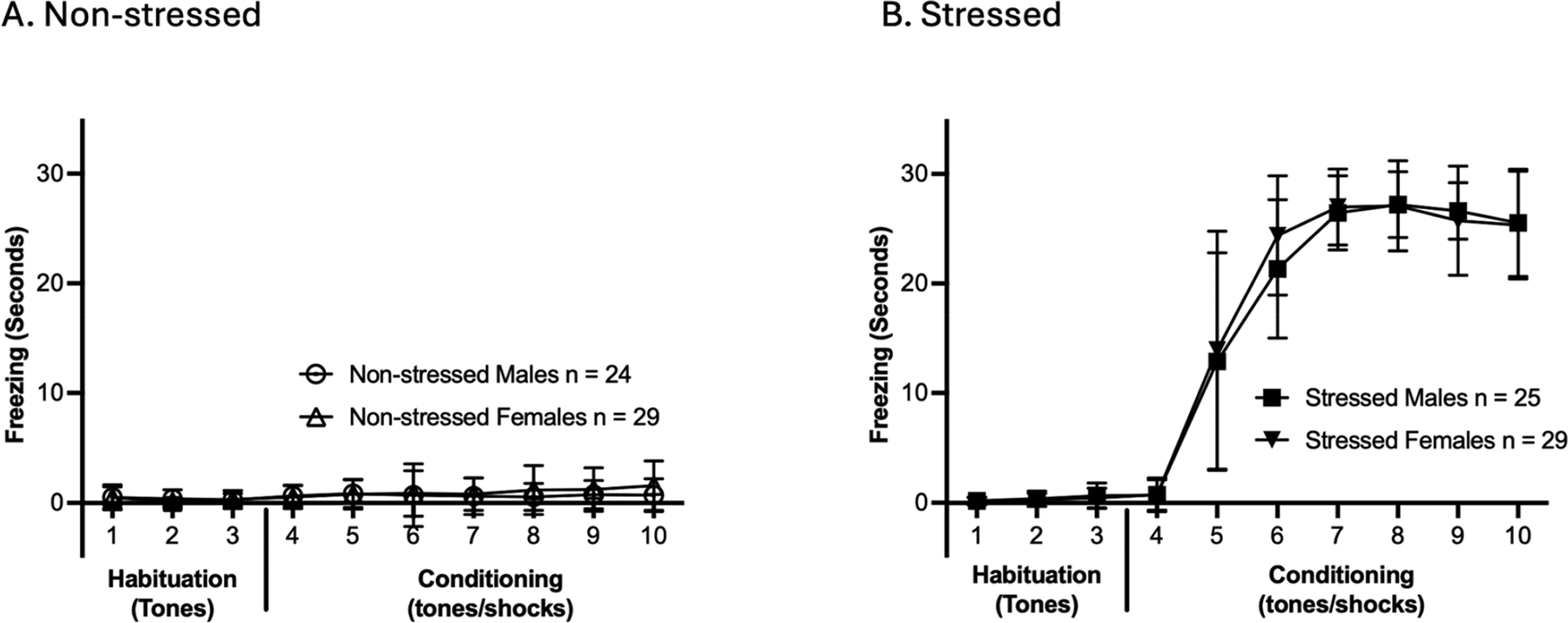
Sex-based comparisons on threat conditioning and tone and context exposure show no difference between groups. (**A**) Freezing during tone presentation in non-stressed rats that received tone and context exposure. (**B**) Freezing during tone presentation in stressed rats that received auditory threat conditioning.

To assess potential sex differences in freezing behavior prior to cocaine or sucrose SA, stressed males and stressed females, as well as their non-stressed counterparts, were compared on TC (Figure 6). There was a significant main effect of time in both the non-stressed male vs female group comparison [F(4.817, 245.7) = 2.308, *p* = 0.0471; (Figure 6A)] and the stressed male vs female group comparison [F(2.165, 112.6) = 450.5, *p* < 0.0001; (Figure 6B)]. Both sexes had similar freezing responses on TC.

### 3.6. Cocaine and Sucrose Self-Administration—Sex Comparisons

**Figure 7.**
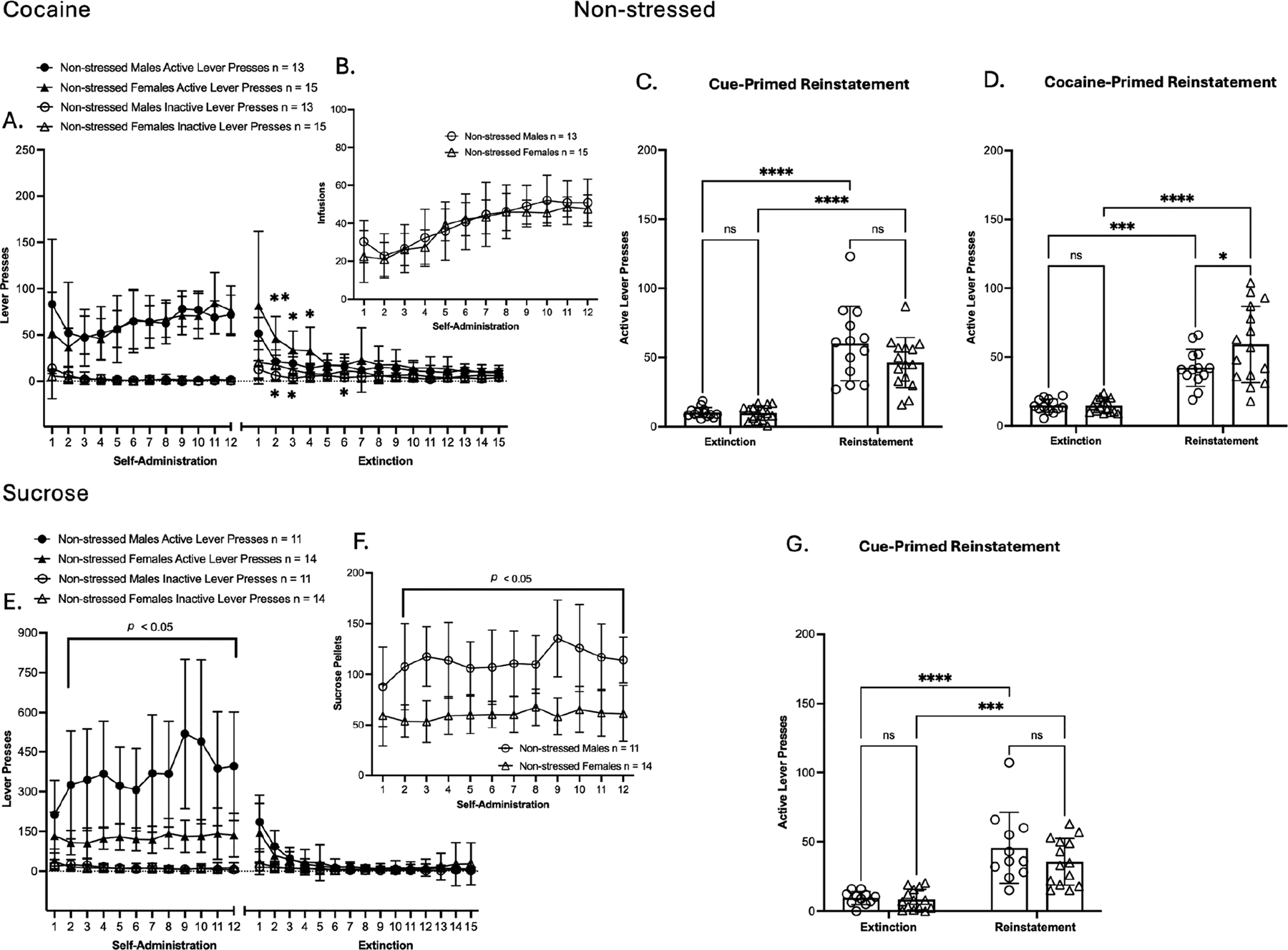
Sex-based comparisons of self-administration revealed that, within the cocaine group, non-stressed females displayed higher extinction lever pressing and greater cocaine-primed reinstatement than non-stressed males. In contrast, non-stressed males in the sucrose group exhibited increased active lever presses and higher sucrose pellet delivery compared to non-stressed females. (**A**) Lever presses during cocaine self-administration and extinction training for non-stressed rats across groups. (**B**) Cocaine infusions in non-stressed rats across groups. (**C**) Reinstatement in the non-stressed group following presentation of cocaine-paired cues, measured by active lever presses (right) and average lever presses during the last three days of extinction training for each group (left). (**D**) Reinstatement in the non-stressed group following cocaine injection, measured by active lever presses (right) and average lever presses during the last two days of extinction training for each group (left). (**E**) Lever presses during sucrose self-administration and extinction training for non-stressed rats across groups. (**F**) Sucrose pellet delivery in non-stressed rats across groups. (**G**) Reinstatement in the non-stressed group following presentation of sucrose-paired cues, measured by active lever presses (right) and average lever presses during the last three days of extinction training for each group (left). *, **, ***, **** Indicate statistical significance shown by post hoc test (*p* < 0.05, 0.01, 0.001, 0.0001, respectively). ns Indicates the non-statistical difference.

**Figure 8.**
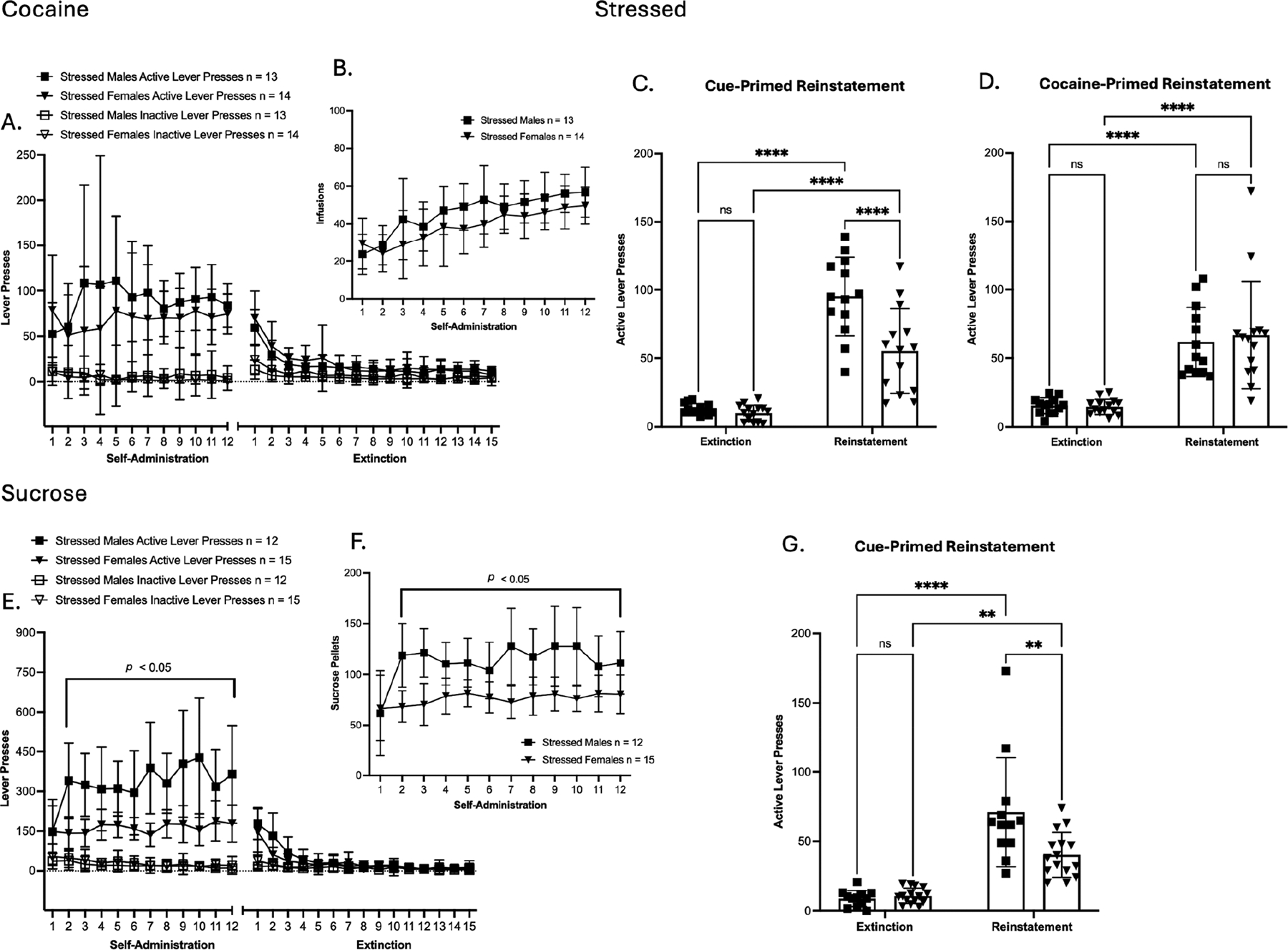
Sex-based comparisons of self-administration indicate that stressed males exhibited higher cue-primed reinstatement than stressed females in both the cocaine and sucrose groups. Additionally, in the sucrose group, stressed males demonstrated greater active lever presses and increased sucrose pellet delivery compared to stressed females. (**A**) Lever presses during cocaine self-administration and extinction training for stressed rats across groups. (**B**) Cocaine infusions in stressed rats across groups. (**C**) Reinstatement in the stressed group following presentation of cocaine-paired cues, measured by active lever presses (right), and the average number of lever presses during the last three days of extinction training for each group (left). (**D**) Reinstatement in the stressed group following cocaine injection, measured by active lever presses (right) and the average number of lever presses during the last two days of extinction training for each group (left). (**E**) Lever presses during sucrose self-administration and extinction training for stressed rats across groups. (**F**) Sucrose pellet delivery in stressed rats across groups. (**G**) Reinstatement in the stressed group following presentation of sucrose-paired cues, measured by active lever presses (right) and the average number of lever presses during the last three days of extinction training for each group (left). **, **** Indicate statistical significance shown by post hoc test (*p* < 0.01, 0.0001). ns Indicates the non-statistical difference.

Sex comparisons were conducted for both cocaine and sucrose SA groups under non-stressed and stressed conditions (Figures 7 and 8). In the following paragraphs, results are presented separately for each of these two conditions.

In the non-stressed cocaine groups, SA active and inactive lever presses showed a significant main effect of time [F(3.176, 82.57) = 5.286, *p* = 0.0018; [F(1.477, 38.40) = 3.831, *p* = 0.0422, respectively (Figure 7A)]. In reward delivery, a significant main effect of time was observed [F(3.829, 99.55) = 42.56, *p* < 0.0001; (Figure 7B)]. SA extinction training active lever presses showed significant main effects of time and sex [F(2.088, 54.29) = 16.25, *p* < 0.0001; F(1, 26) = 5.281, *p* = 0.0299; (Figure 7A)]. Post hoc comparisons indicated that non-stressed females had significantly higher active lever presses than non-stressed males during cocaine SA extinction on days 2–4 (* = *p* < 0.05, uncorrected Fisher’s LSD test; Figure 7A). Extinction training inactive lever presses showed significant main effects of time and sex [F(4.305, 111.9) = 5.593, *p* = 0.0003; F(1, 26) = 6.491, *p* = 0.0171; (Figure 7A)]. Post hoc comparisons indicated that non-stressed females had significantly higher inactive lever presses than non-stressed males during cocaine SA extinction on days 2, 3, and 6 (* = *p* < 0.05, uncorrected Fisher’s LSD test; Figure 7E). Bodyweight % change across SA showed significant main effects of time, sex, and time × sex interaction [F(2.429, 63.15) = 151.3, *p* < 0.0001; F(1, 26) = 32.87, *p* < 0.0001; F(2.429, 63.15) = 23.36, *p* < 0.0001; (Figure S3A)]. Taken together, these results show that, under non-stressed conditions, males and females had similar cocaine SA across sessions. However, during the early phase of cocaine SA extinction, non-stressed female rats had a slower initial reduction in active lever response compared to non-stressed male rats. Active lever presses decreased across cocaine SA extinction sessions, indicating successful extinction in both sexes. Importantly, a main effect of sex was observed, with females exhibiting significantly higher active lever presses than males across extinction. In addition, males had a higher bodyweight % increase than females, showing a sex-specific difference. In cue-primed reinstatement, a significant main effect of time was observed in active lever presses compared to the average of the last three days of extinction [F(1, 52) = 97.31, *p* < 0.0001; (Figure 7C)]. However, within the cue-primed reinstatement groups, no differences were observed in active lever presses between non-stressed groups. In cocaine-primed reinstatement, results showed a significant main effect of time in active lever presses compared to the average of the last two days of post-cue extinction sessions [F(1, 52) = 69.39, *p* < 0.0001; (Figure 7D)]. Post hoc tests showed that non-stressed females had higher cocaine-primed reinstatement than non-stressed males (*p* = 0.0387, Tukey’s multiple comparisons test). These results show that, under non-stressed conditions, reinstatement was influenced by sex in the cocaine SA group only, as females had greater cocaine-primed reinstatement than males.

In the non-stressed sucrose groups, SA active lever presses showed significant main effects of time, sex, and time × sex interaction [F(3.869, 88.98) = 4.814, *p* = 0.0016; F(1, 23) = 26.37, *p* < 0.0001; F(3.869, 88.98) = 4.202, *p* = 0.0040; (Figure 7E)]. Post hoc comparisons confirmed that non-stressed males had significantly higher active lever presses than non-stressed females during sucrose SA on days 2–12 (* = *p* < 0.05, uncorrected Fisher’s LSD test; Figure 7E). SA inactive lever presses showed a significant main effect of time [F(3.828, 88.05) = 5.175, *p* = 0.0010; (Figure 7E)]. In reward delivery, a significant main effect of sex was observed [F(1, 23) = 41.76, *p* < 0.0001; (Figure 7F)]. Post hoc comparisons confirmed that non-stressed males had significantly higher sucrose pellet delivery than non-stressed females during sucrose SA on days 2–12 (* = *p* < 0.05, uncorrected Fisher’s LSD test; Figure 7F). In SA extinction training, active lever presses showed a significant main effect of time [F(2.386, 54.88) = 36.23, *p* < 0.0001; (Figure 7E)], but no significant effects were observed in extinction training inactive lever presses. Bodyweight % change across SA showed significant main effects of time, sex, and time × sex interaction [F(4.468, 102.8) = 373.0, *p* < 0.0001; F(1, 23) = 35.71, *p* < 0.0001; F(4.468, 102.8) = 12.18, *p* < 0.0001; (Figure S3B)]. Taken together, these results show that, under non-stressed conditions, males had higher active lever pressing and sucrose pellet delivery compared to females, suggesting higher motivation for non-drug rewards in males. In addition, non-stressed males of both the cocaine and sucrose SA groups had a higher bodyweight % increase than non-stressed females, showing a sex-specific difference regardless of reward type. In cue-primed reinstatement testing, a significant main effect of time was observed in active lever presses compared to the average of the last three days of extinction [F(1, 46) = 50.83, *p* < 0.0001; (Figure 7G)]. However, within the cue-primed reinstatement groups, no differences were observed in active lever presses between non-stressed groups. These results show that, under non-stressed conditions, reinstatement was not influenced by prior TC in the sucrose groups.

In the stressed cocaine groups, neither SA active lever presses nor inactive lever presses showed any significant effects (Figure 8A). In reward delivery, a significant main effect of time was observed [F(3.806, 95.15) = 23.26, *p* < 0.0001; (Figure 8B)]. SA extinction training active lever presses showed a significant main effect of time [F(4.644, 116.1) = 31.69, *p* < 0.0001; (Figure 8A)]. Extinction training inactive lever presses showed a significant main effect of time [F(4.672, 116.8) = 9.789, *p* < 0.0001; (Figure 8A)]. Bodyweight % change across SA showed significant main effects of time, sex, and time × sex interaction [F(5.800, 145.0) = 260.3, *p* < 0.0001; F(1, 25) = 177.3, *p* < 0.0001; F(5.800, 145.0) = 85.77, *p* < 0.0001; (Figure S3C)]. In general, these results show that, under stressed conditions, males and females had similar cocaine SA and extinction across sessions. In addition, males had a higher bodyweight % increase than females, showing a sex-specific difference. In cue-primed reinstatement, a significant main effect of time was observed in active lever presses compared to the average of the last three days of extinction [F(1, 50) = 118.9, *p* < 0.0001; (Figure 8C)]. In addition, within cue-primed reinstatement groups, significant main effects of treatment and time × treatment interaction were observed [F(1, 50) = 13.58, *p* = 0.0006; F(1, 50) = 9.927, *p* = 0.0027; (Figure 8C)]. Post hoc tests confirmed that stressed males exhibited greater cue-primed reinstatement than stressed females (*p* < 0.0001, Tukey’s multiple comparisons test). In cocaine-primed reinstatement, results showed a significant main effect of time in active lever presses compared to the average of the last two days of post-cue extinction sessions [F(1, 50) = 57.93, *p* < 0.0001; (Figure 8D)]. However, within the cocaine-primed reinstatement groups, no differences were observed in active lever presses between stressed groups. In summary, under stressed conditions, there were sex-specific differences in cue-primed reinstatement only, as males had greater cue-induced cocaine seeking than females.

In the stressed sucrose groups, SA active lever presses showed significant effects of time, sex, and time × sex interaction [F(5.560, 139.0) = 5.341, *p* < 0.0001; F(1, 25) = 27.41, *p* < 0.0001; F(5.560, 139.0) = 4.946, *p* = 0.0002; (Figure 8E)]. Post hoc comparisons confirmed that stressed males had significantly higher active lever presses than stressed females during sucrose SA on days 2–12 (* = *p* < 0.05, uncorrected Fisher’s LSD test; Figure 8E). SA inactive lever presses showed a significant main effect of time [F(4.438, 110.9) = 4.972, *p* = 0.0007; (Figure 8E)]. In reward delivery, significant main effects of time, sex, and time × sex interaction were observed [F(4.692, 117.3) = 7.824, *p* < 0.0001; F(1, 25) = 27.51, *p* < 0.0001; F(4.692, 117.3) = 5.343, *p* = 0.0003; (Figure 8F)]. Post hoc comparisons confirmed that stressed males had significantly higher sucrose pellet delivery than stressed females during sucrose SA on days 2–12 (* = *p* < 0.05, uncorrected Fisher’s LSD test; Figure 8F). SA extinction training active lever presses showed significant main effects of time and time–sex interaction [F(3.074, 76.84) = 55.96, *p* < 0.0001; F(3.074, 76.84) = 3.649, *p* = 0.0155; (Figure 8E)]. Extinction training inactive lever presses showed a significant main effect of time [F(4.975, 124.4) = 3.741, *p* = 0.0035; (Figure 8E)]. Bodyweight % change across SA showed significant main effects of time, sex, and time × sex interaction [F(2.801, 70.02) = 285.6, *p* < 0.0001; F(1, 25) = 11.46, *p* = 0.0023; F(2.801, 70.02) = 9.820, *p* < 0.0001; (Figure S3D)]. In general, these results show that, under stressed conditions, sex influenced sucrose SA, as males had higher active lever pressing and sucrose pellet delivery compared to females, showing a higher motivation for non-drug rewards in males. In addition, stressed males of both cocaine and sucrose SA groups had a higher bodyweight % increase than stressed females, showing a sex-specific difference regardless of reward type or treatment. In cue-primed reinstatement, a significant main effect of time was observed in active lever presses compared to the average of the last three days of extinction [F(1, 50) = 65.31, *p* < 0.0001; (Figure 8G)]. In addition, within cue-primed reinstatement groups, significant main effects of treatment and time × treatment interaction were observed [F(1, 50) = 6.475, *p* = 0.0141; F(1, 50) = 8.283, *p* = 0.0059; (Figure 8G)]. Post hoc tests confirmed that stressed males had greater cue-primed reinstatement than stressed females (*p* = 0.0019, Tukey’s multiple comparisons test).

In summary, under stressed conditions, there were sex-specific differences in cue-primed reinstatement only, regardless of reward type, as males had greater cue-induced cocaine and sucrose seeking than females.

### 3.7. Threat Extinction and Extinction Retrieval—Sex Comparisons

**Figure 9.**
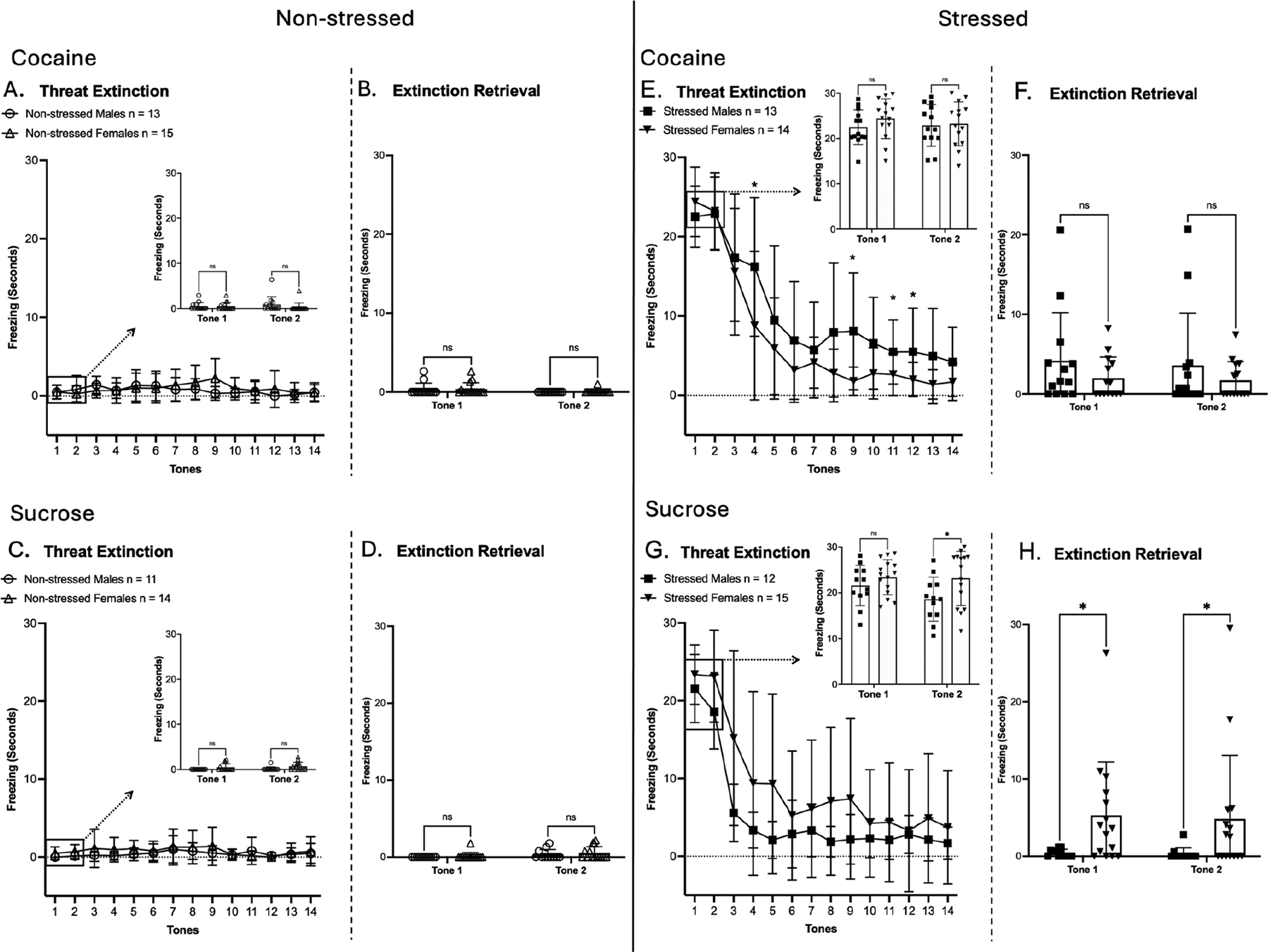
Sex-based comparisons on threat extinction, threat conditioning retrieval, and extinction retrieval show that stressed males had higher freezing than stressed females in threat extinction for the cocaine groups. Conversely, in the sucrose groups, stressed females had higher freezing than stressed males in threat conditioning retrieval and extinction retrieval. (**A**) Threat extinction (day 53) of the non-stressed cocaine group. The first two tones of the threat extinction of the non-stressed cocaine group represent threat conditioning retrieval. (**B**) Extinction retrieval (day 54) of the non-stressed cocaine group. (**C**) Threat extinction (day 42) of the non-stressed sucrose group. The first two tones of the threat extinction of the non-stressed sucrose group represent threat conditioning retrieval. (**D**) Extinction retrieval (day 43) of the non-stressed sucrose group. (**E**) Threat extinction (day 53) of the stressed cocaine group. The first two tones of the threat extinction of the stressed cocaine group represent threat conditioning retrieval. (**F**) Extinction retrieval (day 54) of the stressed cocaine group. (**G**) Threat extinction (day 42) of the stressed sucrose group. The first two tones of the threat extinction of the stressed sucrose group represent threat conditioning retrieval. (**H**) Extinction retrieval (day 43) of the stressed sucrose group. * Indicates statistical significance shown by post hoc test (*p* < 0.05). ns Indicates the non-statistical difference.

To examine whether sex influenced extinction learning and retrieval, cocaine and sucrose groups were compared in terms of freezing behavior during threat extinction, TC retrieval, and extinction retrieval (Figure 9).

In the non-stressed groups, there were no differences in threat extinction or TC retrieval in either the cocaine SA group or the sucrose SA group (Figure 9A,C). At extinction retrieval, a significant main effect on time was shown in both the cocaine SA group [F(1, 26) = 5.619, *p* = 0.0255; (Figure 9B)] and the sucrose SA group [F(1, 23) = 7.315, *p* = 0.0126; (Figure 9D)]. In general, results show that, under non-stressed conditions, males and females exhibited similar freezing during threat extinction, TC retrieval, and extinction retrieval in both the cocaine and sucrose groups.

In the stressed cocaine group, threat extinction showed significant main effects of time and sex [F(5.143, 128.6) = 64.25, *p* < 0.0001; F(1, 25) = 5.263, *p* = 0.0305; (Figure 9E)]. Post hoc comparisons confirmed that stressed males had significantly higher freezing during threat extinction compared to stressed females during tones 4, 9, 11, and 12 (* = *p* < 0.05, uncorrected Fisher’s LSD test; Figure 9E). In TC and extinction retrievals, there were no significant effects (Figure 9E, insert; Figure 9F).

In the stressed sucrose group, threat extinction showed significant main effects of time [F(5.112, 127.8) = 43.71, *p* < 0.0001; (Figure 9G)]. In TC retrieval, a significant main effect of sex was observed [F(1, 25) = 4.351, *p* = 0.0473; (Figure 9G, insert)], with stressed females exhibiting higher freezing than stressed males. At extinction retrieval, a significant main effect of sex was observed [F(1, 25) = 5.051, *p* = 0.0337; (Figure 9H)], showing that stressed females maintained higher freezing compared to stressed males.

Taken together, results show that, under stressed conditions, sex differences emerged in specific phases of extinction and retrieval depending on the type of reward. Overall, these findings reveal that TC exposure affected extinction and retrieval processes in a sexand reward-dependent manner, with females showing more persistent freezing behavior in sucrose groups and males showing higher but more transient freezing responses in cocaine groups.

### 3.8. Open Field Test Sex Comparisons of Cocaine Self-Administration Groups

**Figure 10.**
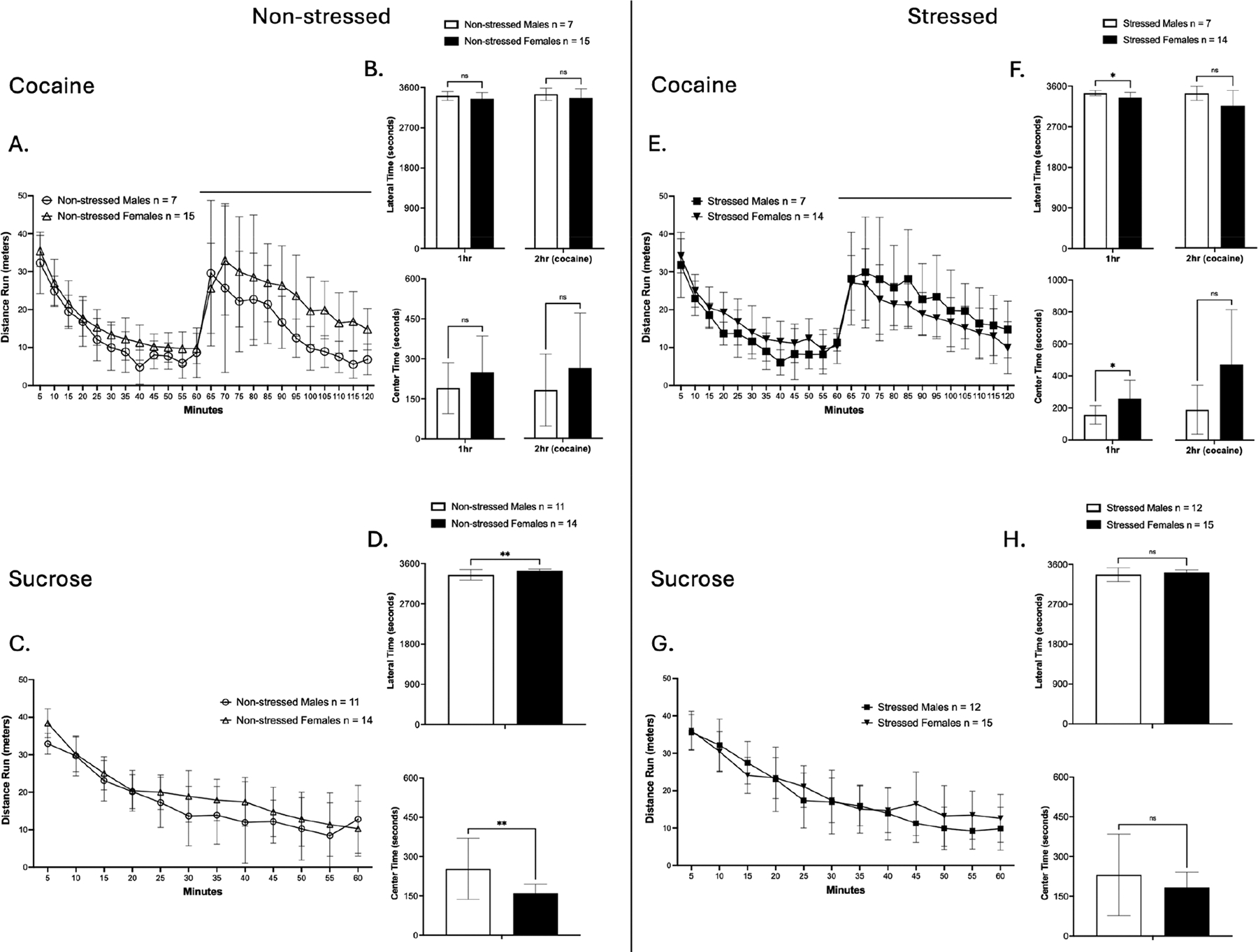
Sex-based comparisons of the cocaine groups showed no differences in locomotor activity or anxiety-like behavior between groups, whereas stressed males show higher anxiety-like behavior than stressed females at baseline without differences in locomotor activity between groups. Sex comparisons in the sucrose groups show that non-stressed females had higher anxiety-like behavior compared to non-stressed males. (**A**) Non-stressed cocaine group, open field total distance divided into 5 min bins across baseline (first 60 min) and after cocaine (10 mg/kg/mL) IP injection (last 60 min), represented by the black bar. (**B**) Non-stressed cocaine group, open field lateral time and center time across baseline (1 h) and after cocaine (10 mg/kg/mL) IP injection (2 h). (**C**) Non-stressed sucrose group, open field total distance divided into 5 min bins. (**D**) Non-stressed sucrose group, open field lateral time and center time. (**E**) Stressed cocaine group, open field total distance divided into 5 min bins across baseline (first 60 min) and after cocaine (10 mg/kg/mL) IP injection (last 60 min), represented by the black bar. (**F**) Stressed cocaine group, open field lateral time and center time across baseline (1 h) and after cocaine (10 mg/kg/mL) IP injection (2 h). (**G**) Stressed sucrose group, open field total distance divided into 5 min bins. (**H**) Stressed sucrose group, open field lateral time and center time. *, ** Indicates statistical significance shown by post hoc test (distance) or *t*-test analysis (lateral and center time) (*p* < 0.05, 0.01, respectively). ns Indicates the non-statistical difference.

To examine whether sex influenced open field behaviors, cocaine and sucrose groups were compared to assess sex differences in locomotor activity and anxiety-like behavior (Figure 10).

In the non-stressed cocaine SA group, a significant main effect of time was observed in locomotor activity in both the first hour [F(6.221, 124.4) = 96.33, *p* < 0.0001; (Figure 10A)] and the second hour [F(2.521, 50.41) = 16.66, *p* < 0.0001; (Figure 10A)]. When assessing cocaine-induced hyperlocomotion, there was a significant main effect of time observed [F(1, 40) = 34.80, *p* < 0.0001; (Figure S4)]. Post hoc tests confirmed that both non-stressed males and non-stressed females displayed cocaine-induced hyperlocomotion (*p* = 0.0094, *p* < 0.0001; Tukey’s multiple comparison test). However, no differences were observed between non-stressed groups in cocaine-induced hyperlocomotion. Anxiety-like behavior showed no significant differences between sexes during either the first hour or the second hour (Figure 10B).

In the non-stressed sucrose SA group, a significant main effect of time was observed [F(5.609, 129.0) = 68.57, *p* < 0.0001; (Figure 10C)], showing no overall sex differences in locomotor activity. Anxiety-like behavior analysis showed a significant difference between sexes [t(23) = 2.828, *p* = 0.0095], with non-stressed females spending more time in the lateral zones compared to males (Figure 10D). In general, no differences were observed in locomotor activity. However, females demonstrated greater anxiety-like behavior, reflected by increased time spent in the lateral zones in the sucrose group only, indicating sex- and reward-dependent differences in anxiety-like behavior.

In the stressed cocaine SA group, a significant main effect of time was observed in locomotor activity in both the first hour [F(5.616, 106.7) = 66.03, *p* < 0.0001; (Figure 10E)] and the second hour [F(5.039, 95.75) = 16.26, *p* < 0.0001; (Figure 10E)]. When assessing cocaine-induced hyperlocomotion, there was a significant main effect of time observed [F(1, 38) = 54.23, *p* < 0.0001; (Figure S4)]. Post hoc test confirmed that both stressed males and stressed females displayed cocaine-induced hyperlocomotion (*p* < 0.0001; Tukey’s multiple comparison test). However, no differences were observed between stressed groups in cocaine-induced hyperlocomotion. Anxiety-like behavior analysis revealed a significant difference during the first hour [t(19) = 2.136, *p* = 0.0459; (Figure 10F)], with stressed males spending more time in the lateral zones compared to stressed females, while no differences were observed during the second hour.

In the stressed sucrose SA group, a significant main effect of time was observed [F(6.510, 162.7) = 77.83, *p* < 0.0001; (Figure 10G)], showing no sex differences in locomotor activity. Anxiety-like behavior analysis showed no significant differences between stressed males and females, either (Figure 10H).

In summary, under stressed conditions, locomotor activity did not differ between males and females in either the cocaine or sucrose groups. Notably, sex differences in anxiety-like behavior emerged during the first hour of the open field test in the cocaine SA group, with stressed males exhibiting a greater lateral zone preference than females, indicating sex-specific anxiety-like responses following TC exposure. In contrast, no sex differences were observed in the sucrose SA groups, suggesting that TC exposure differentially modulated sex-related differences in initial anxiety-like behavior, depending on the reward type.

## 4. Discussion

### 4.1. Influence of Traumatic Stress on Cocaine-Seeking Behavior

The present study investigated how exposure to a traumatic event through threat conditioning (TC) influences cocaine-seeking behavior in a preclinical rodent model. Consistent with prior research, which shows that stress can enhance cocaine-seeking behavior, we found that TC increased cue- and cocaine-primed reinstatement in male rats. In contrast, females did not exhibit a stress-induced increase in cocaine-seeking behavior. These findings suggest that traumatic event exposure can differentially contribute to substance use vulnerability in a sex-dependent manner.

### 4.2. Pavlovian Threat Conditioning as a Stressor

It is essential to select stress protocols that closely replicate the specific trauma being modeled, as different stressors can differentially impact cocaine self-administration outcomes. For instance, restraint stress, designed to mimic psychological stress, has been shown to increase the acquisition of cocaine self-administration [16]. Similarly, early-life exposure to limited bedding and nesting, which simulates early-life adversity, also leads to an increase in the acquisition of cocaine self-administration [21]. In another study, rats that experienced a single exposure to the fox pheromone 2,5-dihydro-2,4,5-trimethylthiazoline (TMT), which imitates innate fear presentation, exhibited impaired extinction learning and increased cue-primed reinstatement [4]. Consistent with prior evidence of stress-induced increases in cocaine-seeking behavior, our laboratory has previously reported that chronic stress exposure in the form of inescapable footshocks prior to cocaine self-administration produces sex-specific differences in the incubation of cocaine cravings, with stressed females displaying greater cue-induced seeking and stressed males displaying greater cocaine-induced seeking following abstinence [18]. For the present study, we chose TC as our stressor, as this model is commonly used in research to study trauma-related disorders [22–27].

Another advantage of using TC is that, as an associative stressor, it allows for the subsequent assessment of extinction learning and retrieval by presenting the conditioned tone without the shock. This approach facilitates the evaluation of how threat memories are regulated and how cocaine may interfere with these processes, which is not feasible with non-associative stressors. Both sexes exhibited greater freezing during threat extinction compared to non-stressed controls. The findings demonstrate that stressed groups exposed to cocaine or sucrose exhibit increased freezing behavior for more than 39 days following TC, indicating that cocaine exposure did not affect TC memory in either sex. Consistent with previous research, Pavlovian TC memory persists for extended periods, as extinguished threat responses can reemerge after 10 days or more, highlighting its long-term retention [30]. Clinical studies also demonstrate that extinguished threat memories may spontaneously recover, complicating the treatment of phobias and other stress-related disorders [31]. A progressive reduction in freezing was observed across trials, indicating that extinction was not impaired and that neither cocaine nor sucrose exposure interfered with this memory. Notably, stressed males displayed greater freezing than stressed females during extinction training, suggesting that males were more vulnerable to the effects of threat and cocaine exposure. Previous studies have similarly reported that cocaine exposure slows threat extinction learning [32]. To better understand such behavioral patterns, it is important to consider the underlying neural mechanisms shared by TC and cocaine exposure. Both TC and cocaine have been shown to alter the balance of prefrontal cortical circuits that regulate threat expression. The Prelimbic Cortex (PL) facilitates conditioned threat expression, whereas the Infralimbic Cortex (IL) supports extinction by inhibiting threat responses [33,34]. Importantly, cocaine exposure produces opposite physiological effects in these regions, as PL pyramidal neurons exhibit increased excitability, while IL pyramidal neurons show depressed activity following cocaine self-administration and extinction training [35]. This cocaine-induced shift in the PL/IL balance provides a possible explanation for the slower extinction learning observed in our cocaine-exposed stressed males, as increased PL activity and decreased IL inhibitory control could result in persistent threat expression. In summary, prior cocaine exposure appears to sensitize threat-related neural circuitry, increasing freezing responses during extinction without producing complete extinction failure. In contrast, stressed females in the present study did not show extinction impairment, having a more resilient extinction profile compared to stressed males. Importantly, while prior studies examining cocaine-related effects on threat extinction have been limited to male rats, our findings provide novel insight into sex-specific differences by characterizing extinction outcomes in females.

Open field testing also showed sex-specific patterns. In males, stress did not alter locomotor activity or anxiety-like behavior, but stressed males did exhibit a trend of stress-induced locomotor activity, particularly during the second half of the test session, which could be a direct effect of dopaminergic sensitization and increased mesolimbic dopamine transmission [36–38]. In females, stress did not affect locomotor activity but instead decreased anxiety-like behavior under cocaine exposure. Together, these results suggest that, while stress enhances cocaine’s stimulant properties in males, it decreases anxiety-like behavior in females, representing additional sex-dependent patterns. Such distinct outcomes may be indicative of differences in neurobiological pathways, with dopaminergic mechanisms being the ones playing a role in male responses and stress–HPA axis interactions having a more direct influence in female responses.

### 4.3. Mechanisms and Brain Areas Involved in Stress-Induced Cocaine Seeking

To better understand the sex-specific effects observed in our study, it is important to consider the neural mechanisms and circuits through which stress can potentiate cocaine seeking. One such mechanism is heightened corticotropin-releasing factor (CRF) signaling in stress-sensitive regions of the brain. CRF is a neuropeptide released in response to stress that plays an important role in physiological and behavioral stress responses [39–41]. It primarily binds to CRF1 receptors, promoting increased CRF signaling, which has been associated with anxiety-like behavior, potentiation of cocaine’s reinforcing effects, and relapse vulnerability [41–44]. It is also known that CRF affects the hypothalamic–pituitary–adrenal (HPA) axis by stimulating the anterior pituitary to secrete the adrenocorticotropic hormone (ACTH), which promotes the release of glucocorticoids, such as corticosterone (CORT), in rodents [45–47]. TC has been shown to cause a transient increase in corticosterone levels shortly after exposure, but levels have shown to decrease after 24 h [23]. These neuroendocrine changes enhance stress responses and alter activity in brain regions involved in emotion regulation and reward seeking, including the prefrontal cortex, amygdala, and nucleus accumbens (NAc) [41,47,48].

Beyond these stress-related neuroendocrine alterations, cortical circuits involved in executive function and decision-making, such as the medial prefrontal cortex (mPFC), may also contribute to stress-induced cocaine seeking. The PL has been implicated in both trauma-related disorders and cocaine use disorder (CUD) [49]. It plays an important role in threat expression, as studies show increased neuronal activity and an increased firing rate of PL neurons during threat consolidation and expression [50,51]. Furthermore, inactivation of the PL has been shown to reduce threat retrieval [52]. The PL also plays a significant role in modulating cocaine seeking, contributing to memory consolidation and reinstatement [49,53]. Cocaine exposure has been shown to increase PL neuronal activity, c-Fos expression, and Arc gene expression during reinstatement [54,55]. In contrast, inactivation of the PL has been found to reduce reinstatement triggered by cocaine, as well as by cues that are associated with the drug [56]. This pathway is important, as PL projections to the NAc core mediate dopamine-dependent cue-induced seeking [57].

Another brain region that plays an important role in both trauma-related disorders and the associative learning of drug-seeking behavior is the basolateral amygdala (BLA) [58–64], which is involved in the expression, retrieval, and extinction of conditioned threat memories [65,66]. BLA inhibition disrupts both recent and remote threat memory retrieval [66]. The BLA is also involved in cocaine seeking, and its photoinhibition, or selective inhibition of BLA-to-NAc core projections, has been shown to significantly attenuate cocaine reinstatement [62]. Importantly, stress exposure has been shown to not only potentiate cocaine-seeking behavior but to also produce increased BLA neuronal activity, further suggesting that this region is a key player when addressing trauma- and cocaine-related circuits [63,64]. Together, these findings support the idea that overlapping neural circuitry may underlie both fear/threat-related and drug-seeking behaviors. Trauma exposure via TC may potentiate cocaine seeking through alterations in these circuits, thereby increasing relapse vulnerability following traumatic event exposure.

### 4.4. Sex Differences in Stress-Induced Cocaine Self-Administration

Sex differences in cocaine self-administration have been widely reported, although the direction and magnitude of these effects vary across studies. One study found that male rats acquired cocaine SA at a higher rate than females, and they met acquisition criteria in fewer sessions. However, once the criteria were reached, females self-administered more cocaine than males during the early maintenance phase [67]. In contrast, another study reported that female rats acquired cocaine SA more rapidly and at a higher rate than males, subsequently also self-administering more cocaine than males [68]. In addition to acquisition and maintenance, sex differences have also been observed in extinction, as females often show higher responding during extinction, particularly in the early phase of extinction [69]. Consistent with the literature, sex comparisons within non-stressed groups revealed higher active lever responding in females relative to males during extinction, with this difference being most evident during the initial extinction sessions. Together, these findings highlight that sex influences multiple aspects of cocaine-seeking behavior, although the specific behavioral trajectory may depend on experimental conditions.

Consistent with the view that stress can interact with sex to shape relapse vulnerability, our data show that TC prior to cocaine exposure selectively increased cocaine seeking in males but not in females. An explanation for the resilience observed in females may lie in the differential levels of sex hormones, such as estradiol and progesterone, and in molecular and/or physiological mechanisms that connect these hormones to both stress- and cocaine-related behaviors. Estradiol, for example, facilitates cocaine seeking and reinstatement mainly through activation of the estrogen receptor β [70–73]. Conversely, progesterone attenuates cocaine seeking through its conversion into the neuroactive steroid allopregnanolone, which has been shown to decrease dopamine concentrations and potentiate GABAA receptor activation, collectively reducing cocaine reinforcement [74–78]. These mechanisms may explain the absence of a stress-induced increase in female cocaine seeking after TC through protective or compensatory hormonal states that attenuate stress-related drug motivation. In a study conducted by Doncheck and others, which showed that footshocks increased cocaine reinstatement in male rats but not in females, they also observed that CORT potentiating effects on reinstatement were estrous-cycle dependent, specifically occurring during diestrus and proestrus stages only [79]. Collectively, these findings suggest that TC influences mesolimbic and neuroendocrine signaling pathways in males, leading to increased reinforcing effects of cocaine. In contrast, in females, TC appears to modulate affective circuitry, with minimal impact on dopaminergic or neuroendocrine pathways. These contrasting effects elucidate mechanisms by which variations in neurobiological adaptations, including hormone levels, may underlie sex-specific differences in the impact of TC on cocaine-seeking behavior.

Interestingly, sex-specific differences were also observed in bodyweight percentage changes across cocaine self-administration. Specifically, we saw that male rats had greater increases in bodyweight relative to females. This is consistent with prior work that shows that male rats gain more weight and at a faster rate than female rats due to sex hormone effects and differences in metabolic rates [80,81]. However, in our case, these differences were not due to treatment, as no differences were observed between the stressed and non-stressed groups. Such an interpretation of weight changes not resulting from stress is consistent with the existing literature, as previous studies have shown that acute stressors are generally not sufficient to produce long-term alterations in bodyweight [82].

### 4.5. Influence of Traumatic Stress on Non-Drug-Seeking Behavior

To assess how trauma exposure influences non-drug reward seeking, we examined the effects of TC on sucrose-seeking behavior in rats. TC significantly increased sucrose self-administration in females, whereas males did not show stress-induced changes. In addition to these behavioral effects, sex comparisons revealed that males exhibited greater sucrose consumption and bodyweight gains than females, which is consistent with prior work showing that male rats gain weight at a faster rate due to hormonal and metabolic factors [80,81]. As in the cocaine groups, this reflected baseline sex differences rather than stress-induced changes, since no differences in bodyweight were observed between stressed and non-stressed animals. Again, this interpretation aligns with previous studies showing that acute stressors are generally not sufficient to produce long-term alterations in bodyweight [82]. Interestingly, sex comparisons in reinstatement showed that stressed males had higher cue-primed reinstatement compared to stressed female rats. Notably, this finding is consistent with previous work that shows that stress in the form of social isolation in male rats increases cue- but not reward-induced reinstatement, suggesting that stress can induce cue-driven relapse-like behavior increases [83].

Overall, our results indicate that trauma exposure can differentially affect non-drug reward seeking in a sex-dependent manner, with stressed females showing heightened sucrose motivation, while bodyweight differences reflect biological sex differences rather than stress effects. When considered in the context of prior research, the literature on stress and sucrose intake has reported mixed outcomes depending on the stress paradigm and timing of exposure. For example, chronic, mild, unpredictable stress has been shown to increase sucrose binge eating and anxiety-like behavior in susceptible rats [84]. In contrast, other research has demonstrated that four weeks of chronic, mild stress in male rats reduced sucrose intake, while also producing marked alterations in sleep [85]. Furthermore, a separate study found that early life stress, in the form of limited nesting, did not alter sucrose consumption in females, suggesting that not all stressors equally impact palatable food intake [86]. Taken together, these contrasting outcomes demonstrate that stress-induced changes in non-drug reward seeking vary across stress modes, reinforcing the importance of examining these effects using TC as an associative stress paradigm.

We also examined how trauma exposure influenced TC memory retrieval and threat extinction in sucrose groups. Stressed males initially showed higher freezing than non-stressed males during threat extinction, but their freezing levels normalized by the end of both protocols, indicating successful extinction learning. In contrast, stressed females exhibited higher freezing across threat extinction and extinction retrieval compared to non-stressed females, showing a more persistent conditioned threat response. When assessing TC memory retrieval, we also observed that stressed groups were higher compared to their non-stressed counterparts, regardless of sex and previous sucrose exposure. This shows that TC memory was still present even past 26 days of TC. Sex comparisons also showed that stressed females maintained higher freezing than stressed males at both TC retrieval and extinction retrieval. These findings differ from the cocaine groups, where stressed males showed greater vulnerability, which could suggest that, when paired with a non-drug reward, stress may prolong threat memory expression to a greater extent in females. These results also highlight that cocaine exposure may alter male threat extinction, resulting in the sex-specific extinction outcomes previously discussed.

Open field testing in sucrose groups revealed no stress-induced differences in locomotor activity or anxiety-like behavior within either sex. However, sex comparisons showed that, under non-stressed conditions, females displayed greater locomotor activity during the initial five minutes of the session and increased anxiety-like behavior, as indicated by more time spent in the lateral zones, compared to males. These findings are consistent with prior work reporting that females generally exhibit higher baseline locomotor activity and greater anxiety-like behavior than males [87,88]. In contrast, these sex differences were absent in stressed groups, suggesting that stress exposure may have increased male anxiety-like behavior to levels comparable to females. Taken together, these results indicate that stress normalizes sex-specific anxiety-like patterns in the sucrose group in contrast to the cocaine group, where stress amplified sex differences by enhancing cocaine’s stimulant effects in males and decreasing cocaine’s anxiogenic effects in females.

### 4.6. Mechanisms and Brain Areas Involved in Stress-Induced Increase in Sucrose Self-Administration

Building on these behavioral findings, it is important to consider the neuroendocrine and neural mechanisms that may regulate sucrose-seeking under stress. The stress-driven increase in motivation to consume highly palatable, sweet foods has been linked to neuroendocrine adaptations involving glucocorticoids and relaxin-3 neuropeptide signaling [89–100]. Relaxin-3 activity, which is involved in brain functions, such as learning, memory, and metabolism, has also been linked to stress-induced sucrose overconsumption in female rats, suggesting a potential sex-specific mechanism influencing non-drug reward seeking under stress [100]. Sucrose consumption has also been shown to reduce behavioral and physiological stress responses by attenuating HPA axis activation and reducing anxiety-like behavior [101–103].

Repeated non-drug reward, such as sugar intake, has also been shown to alter the mesolimbic dopamine system, particularly NAc, and to promote changes that reinforce craving and seeking behavior [104–106]. In addition to reward-related structures, other structures such as the amygdala, prefrontal cortex, and anterior hypothalamic area have also been shown to be involved in emotional regulation, behavioral flexibility, feeding behavior, and stress response, which become increasingly sensitized under stress and can contribute to increased non-drug reward seeking following threat exposure [106,107].

### 4.7. Limitations

While our study provides important insights into how TC influences cocaine- and sucrose-seeking behavior, several limitations should be considered when interpreting these findings. Neurobiological and neuroendocrine mechanisms were inferred but not directly measured, as no assays of neural activity, molecular analysis, or stress hormone levels were conducted. Including CORT measurements at key time points during the behavioral protocols could also help further explain the effects of TC on both cocaine- and sucrose-seeking behavior. In addition, results derived from TC reflect a specific type of traumatic event, and they might not fully represent the complexity of human trauma, including scenarios in which multiple stressors occur simultaneously.

## 5. Conclusions

This study provides preclinical evidence that traumatic event exposure differentially affects reward-seeking behavior based on sex and the reward type, as TC produced distinct behavioral outcomes in males versus females. Males were more vulnerable to trauma-induced increases in cocaine seeking, whereas females showed greater non-drug reward seeking. Our findings support models proposing that trauma alters reward processing and disrupts the mechanisms regulating motivated behavior in a sex- and reward-dependent manner. These findings highlight the necessity of sex-specific evaluations for individuals when assessing relapse vulnerability and formulating intervention strategies. Future research should assess the function of neural circuits in key brain regions, including the mPFC, NAc, BLA, and ventral tegmental area. Applying advanced approaches, such as c-Fos mapping to identify active neuronal populations, optogenetics for precise manipulation of circuit activity, and in vivo calcium imaging to track real-time neural dynamics, will help clarify the pathways through which trauma modulates reward seeking. These methods may also reveal sex-specific mechanisms, contributing to a more comprehensive understanding of neural function and its implications for behavior and mental health.

## Supplementary Material

Supplementary Material

**Supplementary Materials:** The following supporting information can be downloaded at: https://www.mdpi.com/article/10.3390/psychiatryint7020085/s1; Figure S1: Number of stressed male and female rats classified as darters or non-darters, and as hoppers or non-hoppers; Figure S2: Non-stressed vs stressed bodyweight % change; Figure S3: Sex comparisons of bodyweight % change; Figure S4: Cocaine-induced hyperlocomotion group comparisons; Table S1: Summary of Statistical Analysis: Threat Conditioning; Table S2: Summary of Statistical Analysis: Cocaine Cohort; Table S3: Summary of Statistical Analysis: Sucrose Cohort.

## Data Availability

Data is contained within the article and the Supplementary Materials.

## Abbreviations

The following abbreviations are used in this manuscript:

ACTH: Adrenocorticotropic Hormone
BLA: Basolateral Amygdala
CORT: Corticosterone
CRF: Corticotropin-releasing Factor
CUD: Cocaine Use Disorder
HPA: Hypothalamic-Pituitary-Adrenal
IACUC: Institutional Animal Care and Use Committee
IL: Infralimbic Cortex
MI: Mental Illness
mPFC: Medial Prefrontal Cortex
Nac: Nucleus Accumbens
NIDA: National Institute on Drug Abuse
NIH: National Institute of Health
NSDUH: National Survey on Drug Use and Health
PL: Prelimbic Cortex
SA: Self-Administration
SAMHSA: Substance Abuse and Mental Health Services Administration
SUD: Substance Use Disorder
TC: Threat Conditioning
TMT: 2,5-dihydro-2,4,5-trimethylthiazoline
