# Supplementary material for: Threat Conditioning Prior to Cocaine or Sucrose Exposure Alters Reward-Seeking Behavior in a Sex-Dependent Manner
Source: Psychiatry Int (Basel). Author manuscript; Available in PMC 2026 Jun 5. (PMC13235266; doi:10.3390/psychiatryint7020085)
Supplement: Supplementary Material [file NIHMS2180594-supplement-Supplementary_Material.pdf]

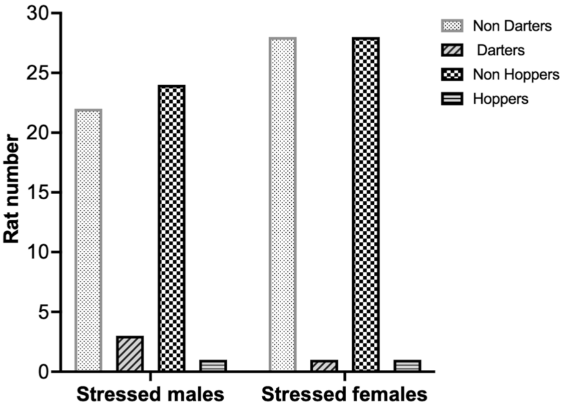

Figure S1: Number of stressed male and female rats classified as darters or non-darters, and as hoppers or non-hoppers, during threat conditioning.

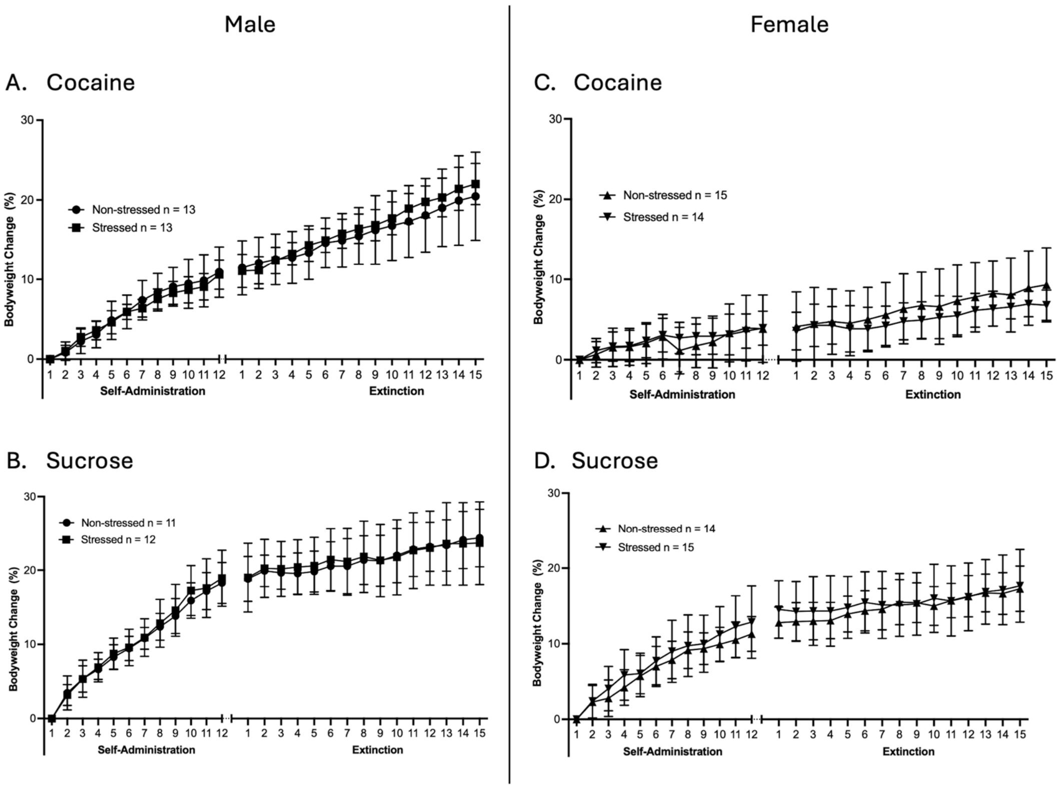

Figure S2: Threat conditioning did not affect bodyweight % change across cocaine or sucrose self-administration in either male or female rats. (A) Bodyweight comparison between non-stressed and stressed males of the cocaine group. (B) Bodyweight comparison between non-stressed and stressed males of the sucrose group. (C) Bodyweight comparison between non-stressed and stressed females of the cocaine group. (D) Bodyweight comparison between non-stressed and stressed females of the sucrose group.

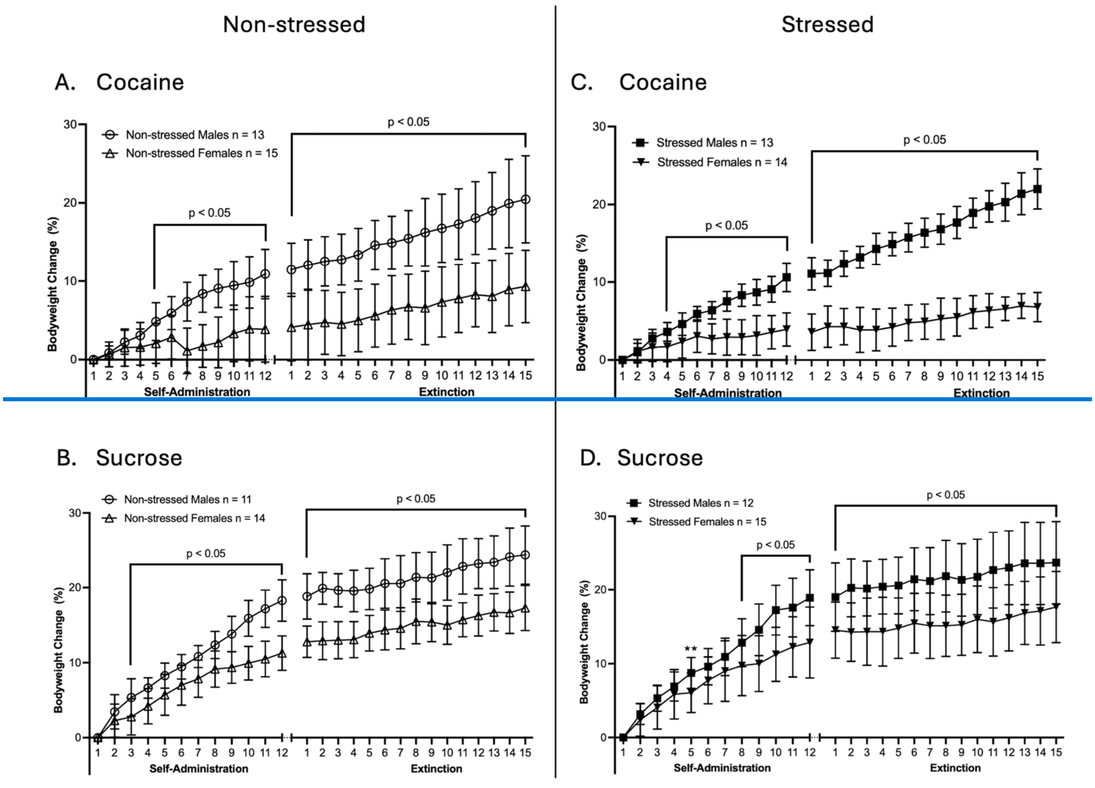
Non-stressed
Stressed
A. Cocaine
Non-stressed Males n = 13
Non-stressed Females n = 15
p < 0.05
p < 0.05
Bodyweight Change (%)
Self-Administration
Extinction
C. Cocaine
Stressed Males n = 13
Stressed Females n = 14
p < 0.05
p < 0.05
Bodyweight Change (%)
Self-Administration
Extinction
B. Sucrose
Non-stressed Males n = 11
Non-stressed Females n = 14
p < 0.05
p < 0.05
Bodyweight Change (%)
Self-Administration
Extinction
D. Sucrose
Stressed Males n = 12
Stressed Females n = 15
p < 0.05
p < 0.05
**
Bodyweight Change (%)
Self-Administration
Extinction

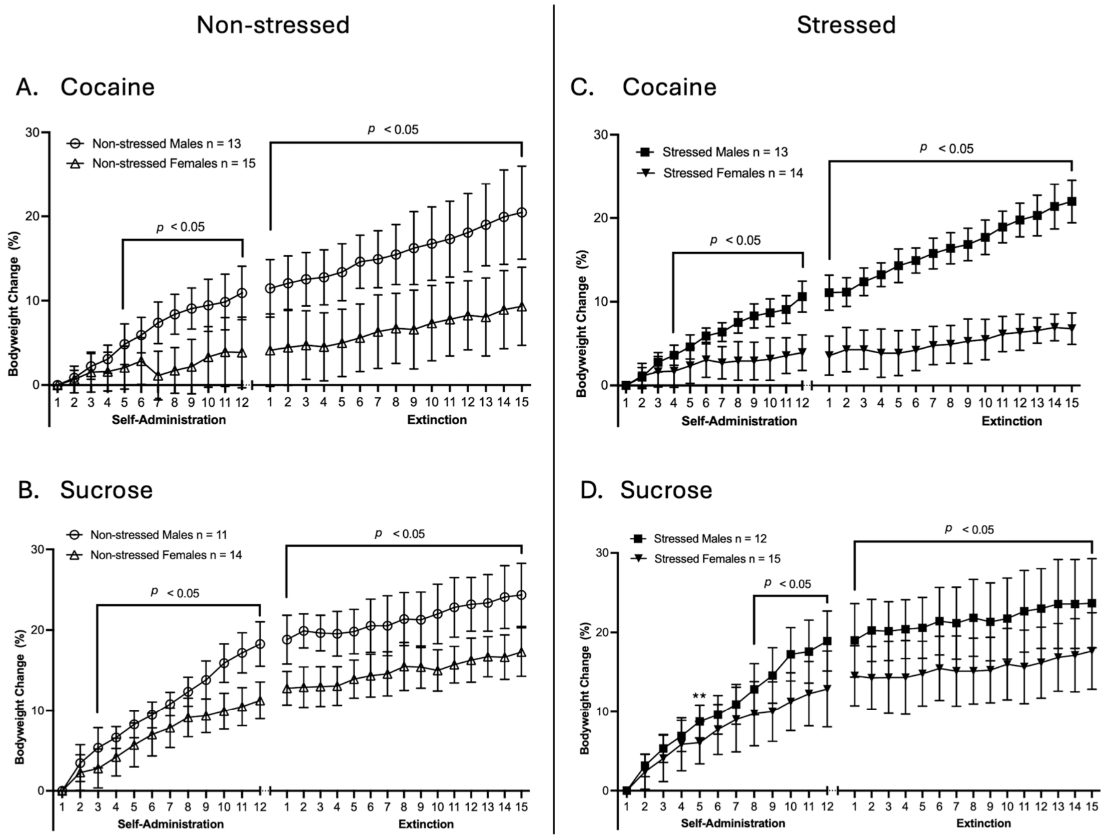
Non-stressed
Stressed
A. Cocaine
Non-stressed Males n = 13
Non-stressed Females n = 15
p < 0.05
p < 0.05
Bodyweight Change (%)
Self-Administration
Extinction
C. Cocaine
Stressed Males n = 13
Stressed Females n = 14
p < 0.05
p < 0.05
Bodyweight Change (%)
Self-Administration
Extinction
B. Sucrose
Non-stressed Males n = 11
Non-stressed Females n = 14
p < 0.05
p < 0.05
Bodyweight Change (%)
Self-Administration
Extinction
D. Sucrose
Stressed Males n = 12
Stressed Females n = 15
p < 0.05
p < 0.05
**
Bodyweight Change (%)
Self-Administration
Extinction

Figure S3: Sex comparisons of bodyweight % change across both cocaine and sucrose self-administration show that male rats had greater body weight gain than females, regardless of group and treatment. (A) Bodyweight comparison between non-stressed males and non-stressed females of the cocaine group. (B) Bodyweight comparison between non-stressed males and non-stressed females of the sucrose group. (C) Bodyweight comparison between stressed males and stressed females of the cocaine group. (D) Bodyweight comparison between stressed males and stressed females of the sucrose group.

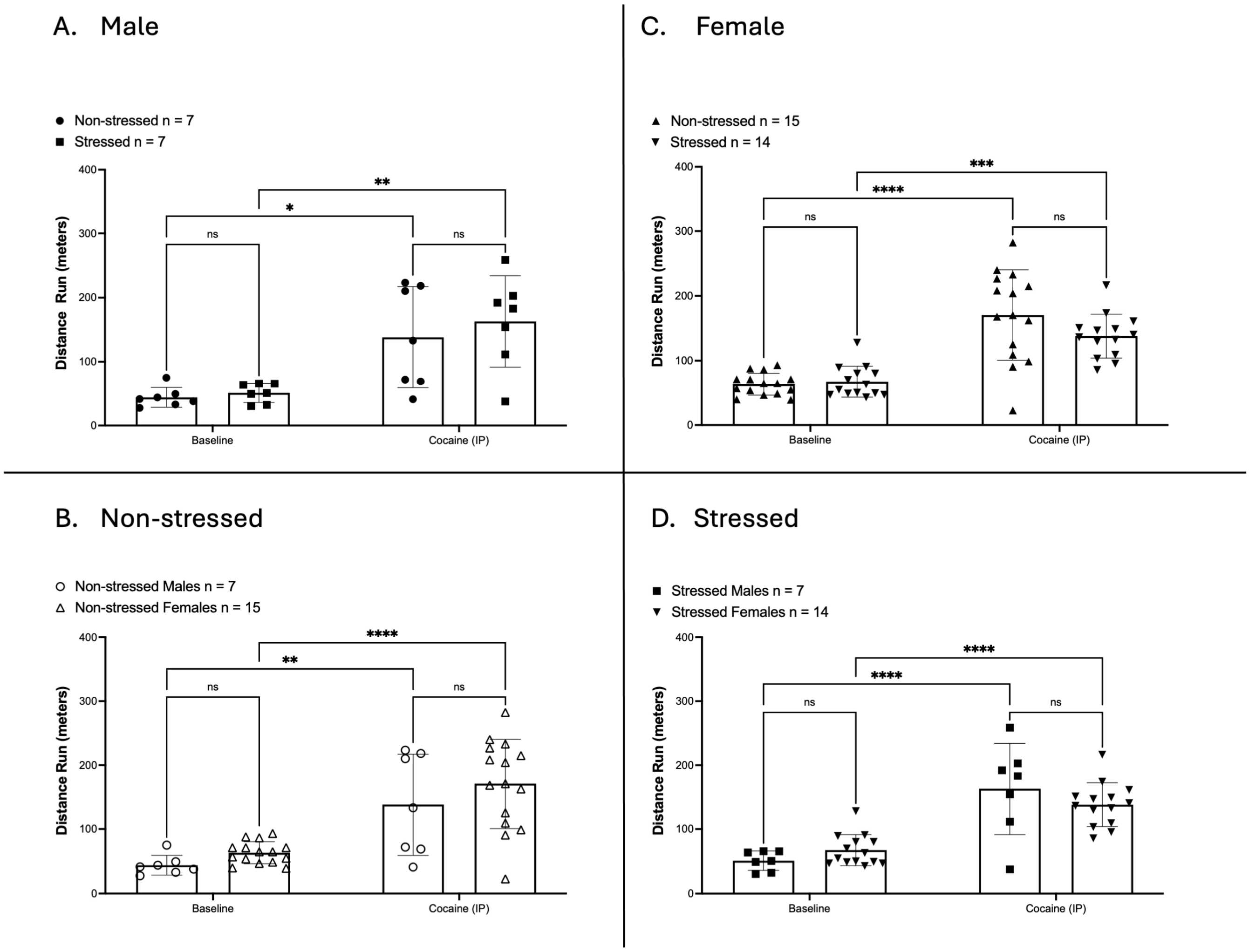

Figure S4: Analysis of cocaine-induced hyperlocomotion in the open field shows cocaine-induced hyperlocomotion in all group comparisons, regardless of treatment or sex. However, results did not show any treatment or sex differences. (A) Locomotor activity comparison between non-stressed males and stressed males. (B) Locomotor activity comparison between non-stressed females and stressed females. (C) Locomotor activity comparison between non-stressed males and non-stressed females. (D) Locomotor activity comparison between stressed males and stressed females. *, **, ***, **** Indicate statistical significance shown by post hoc test ($p < 0.05, 0.01, 0.001, 0.0001$, respectively). ns Indicates the non-statistical difference. ~~*Indicates statistical significance shown by post hoc test (p < 0.05).~~

Table 1: Summary of Statistical Analysis: Threat Conditioning

Behavioral Phase	Comparison	Analysis	Factor	F(df)	*p*-value
Threat Conditioning	Male	Two-way repeated measures ANOVA	**Time**	**F (2.160, 101.5) = 201.3**	**< 0.0001**
			**Treatment**	**F (1, 47) = 1011**	**< 0.0001**
			**Time × Treatment**	**F (2.160, 101.5) = 195.2**	**< 0.0001**
	Female		**Time**	**F (2.528, 141.6) = 222.8**	**<0.0001**
			**Treatment**	**F (1, 56) = 1013**	**<0.0001**
			**Time × Treatment**	**F (2.528, 141.6) = 197.7**	**<0.0001**
	Non-stressed		**Time**	**F (4.817, 245.7) = 2.308**	**0.0471**
			Sex	F (1, 51) = 0.7037	0.4054
			Time × Sex	F (4.817, 245.7) = 0.9035	0.4766

	Stressed		**Time**	**F (2.165, 112.6) = 450.5**	**<0.0001**
			Sex	F (1, 52) = 0.3038	0.5839
			Time × Sex	F (2.165, 112.6) = 0.8329	0.4456

Table 2: Summary of Statistical Analysis: Cocaine Cohort

Behavioral Phase	Comparison	Analysis	Factor	F(df)	*p*-value
Self-Administration Active Lever Presses	Male	Two-way repeated measures ANOVA	Time	F (3.431, 82.34) = 0.9504	0.4293
			Treatment	F (1, 24) = 3.315	0.0811
			Time × Treatment	F (3.431, 82.34) = 2.388	0.0667
	Female		Time	F (2.324, 62.74) = 2.935	0.0528
			Treatment	F (1, 27) = 0.3719	0.5471
			Time × Treatment	F (2.324, 62.74) = 0.7667	0.4867
	Non-stressed		**Time**	**F (3.176, 82.57) = 5.286**	**0.0018**
			Sex	F (1, 26) = 0.1971	0.6607
			Time × Sex	F (3.176, 82.57) = 1.430	0.2386
	Stressed		Time	F (3.322, 83.04) = 1.210	0.3121
			Sex	F (1, 25) = 1.492	0.2334
			Time × Sex	F (3.322, 83.04) = 1.319	0.2729
Self-Administration Inactive Lever Presses	Male	Two-way repeated measures ANOVA	Time	F (2.344, 56.25) = 1.551	0.2181
			Treatment	F (1, 24) = 1.118	0.3009
			Time × Treatment	F (2.344, 56.25) = 0.4555	0.6672
	Female		**Time**	**F (4.602, 124.2) = 8.076**	**<0.0001**
			Treatment	F (1, 27) = 1.927	0.1764
			Time × Treatment	F (4.602, 124.2) = 2.085	0.0772
	Non-stressed		**Time**	**F (1.477, 38.40) = 3.831**	**0.0422**
			Sex	F (1, 26) = 0.2387	0.6292
			Time × Sex	F (1.477, 38.40) = 0.9975	0.3560
	Stressed		Time	F (1.926, 48.15) = 1.442	0.2464
			Sex	F (1, 25) = 0.7307	0.4008
			Time × Sex	F (1.926, 48.15) = 0.6410	0.5255
Extinction Active Lever Presses	Male	Two-way repeated measures ANOVA	**Time**	**F (5.200, 124.8) = 49.82**	**<0.0001**
			Treatment	F (1, 24) = 0.7358	0.3995
			Time × Treatment	F (5.200, 124.8) = 1.003	0.4205
	Female		**Time**	**F (2.679, 72.32) = 19.57**	**<0.0001**
			Treatment	F (1, 27) = 0.8413	0.3671
			Time × Treatment	F (2.679, 72.32) = 0.5187	0.6503
	Non-stressed		**Time**	**F (2.088, 54.29) = 16.25**	**<0.0001**
			**Sex**	**F (1, 26) = 5.281**	**0.0299**
			Time × Sex	F (2.088, 54.29) = 1.876	0.1614
	Stressed		**Time**	**F (4.644, 116.1) = 31.69**	**<0.0001**
			Sex	F (1, 25) = 0.5268	0.4747
			Time × Sex	F (4.644, 116.1) = 1.071	0.3784
Extinction Inactive Lever Presses	Male	Two-way repeated measures ANOVA	**Time**	**F (4.874, 117.0) = 7.790**	**<0.0001**
			Treatment	F (1, 24) = 0.06407	0.8023
			Time × Treatment	F (4.874, 117.0) = 0.7724	0.5685
	Female		**Time**	**F (4.654, 125.6) = 9.184**	**<0.0001**
			Treatment	F (1, 27) = 0.2382	0.6294
			Time × Treatment	F (4.654, 125.6) = 0.6810	0.6282
	Non-stressed		**Time**	**F (4.305, 111.9) = 5.593**	**0.0003**
			**Sex**	**F (1, 26) = 6.491**	**0.0171**
			Time × Sex	F (4.305, 111.9) = 1.422	0.2283
	Stressed		**Time**	**F (4.672, 116.8) = 9.789**	**<0.0001**
			Sex	F (1, 25) = 2.274	0.1441
			Time × Sex	F (4.672, 116.8) = 1.966	0.0936
Self-Administration Cocaine Infusions	Male	Two-way repeated measures ANOVA	**Time**	**F (4.168,100.0) = 28.65**	**< 0.0001**
			Treatment	F (1,24) = 2.083	0.1619
			Time × Treatment	F (4.168,100.0) = 2.094	0.0844
	Female		**Time**	**F (3.225,87.06) = 33.34**	**< 0.0001**

			Treatment	F (1,27) = 0.07601	0.7849
			Time × Treatment	F (3.225,87.06) = 1.141	0.3389
	Non-stressed		**Time**	**F (3.829, 99.55) = 42.56**	**<0.0001**
			Sex	F (1, 26) = 0.5803	0.4530
			Time × Sex	F (3.829, 99.55) = 1.022	0.3977
	Stressed		**Time**	**F (3.806, 95.15) = 23.26**	**<0.0001**
			Sex	F (1, 25) = 3.602	0.0693
			Time × Sex	F (3.806, 95.15) = 1.738	0.1510
Self-Administration Bodyweight %	Male	Two-way repeated measures ANOVA	**Time**	**F (2.472,59.32) = 367.3**	**< 0.0001**
			Treatment	F (1,24) = 0.1090	0.7441
			Time × Treatment	F (2.472,59.32) = 1.760	0.1733
	Female		**Time**	**F (3.879,104.7) = 58.89**	**< 0.0001**
			Treatment	F (1,27) = 0.3621	0.5523
			**Time × Treatment**	**F (3.879,104.7) = 3.343**	**0.0137**
	Non-stressed		**Time**	**F (2.429, 63.15) = 151.3**	**<0.0001**
			**Sex**	**F (1, 26) = 32.87**	**<0.0001**
			**Time × Sex**	**F (2.429, 63.15) = 23.36**	**<0.0001**
	Stressed		**Time**	**F (5.800, 145.0) = 260.3**	**<0.0001**
			**Sex**	**F (1, 25) = 177.3**	**<0.0001**
			**Time × Sex**	**F (5.800, 145.0) = 85.77**	**<0.0001**
Cue-Primed Reinstatement	Male	Two-way ANOVA	**Time**	**F (1,48) = 143.7**	**< 0.0001**
			**Treatment**	**F (1,48) = 11.78**	**0.0012**
			**Interaction**	**F (1,48) = 8.555**	**0.0052**
	Female		**Time**	**F (1,54) = 72.63**	**< 0.0001**
			Treatment	F (1,54) = 0.8757	0.3535
			Interaction	F (1,54) = 0.8689	0.3554
	Non-stressed		**Time**	**F (1, 52) = 97.31**	**<0.0001**
			Sex	F (1, 52) = 2.649	0.1097
			Interaction	F (1, 52) = 2.399	0.1275
	Stressed		**Time**	**F (1, 50) = 118.9**	**<0.0001**
			**Sex**	**F (1, 50) = 13.58**	**0.0006**
			**Time × Sex**	**F (1, 50) = 9.927**	**0.0027**
Cocaine-Primed Reinstatement	Male	Two-way ANOVA	**Time**	**F (1,48) = 80.90**	**< 0.0001**
			**Treatment**	**F (1,48) = 6.076**	**0.0173**
			**Interaction**	**F (1,48) = 5.231**	**0.0266**
	Female		**Time**	**F (1,54) = 58.48**	**< 0.0001**
			Treatment	F (1,54) = 0.3531	0.5548
			Interaction	F (1,54) = 0.3781	0.5412
	Non-stressed		**Time**	**F (1, 52) = 69.39**	**< 0.0001**
			Sex	F (1, 52) = 3.779	0.0573
			Interaction	F (1, 52) = 3.837	0.0555
	Stressed		**Time**	**F (1, 50) = 57.93**	**<0.0001**
			Sex	F (1, 50) = 0.09922	0.7541
			Interaction	F (1, 50) = 0.2096	0.6491
Threat Extinction	Male	Two-way repeated measures ANOVA	**Time**	**F (4.792,115.0) = 21.15**	**< 0.0001**
			**Treatment**	**F (1,24) = 58.54**	**< 0.0001**
			**Time × Treatment**	**F (4.792,115.0) = 19.57**	**< 0.0001**
	Female		**Time**	**F (4.167,112.5) = 45.72**	**< 0.0001**
			**Treatment**	**F (1,27) = 109.6**	**< 0.0001**
			**Time × Treatment**	**F (4.167,112.5) = 49.70**	**< 0.0001**
	Non-stressed		Time	F (7.548, 196.2) = 1.665	0.1136
			Sex	F (1, 26) = 1.019	0.3220
			Time × Sex	F (7.548, 196.2) = 1.618	0.1263
	Stressed		**Time**	**F (5.143, 128.6) = 64.25**	**<0.0001**
			**Sex**	**F (1, 25) = 5.263**	**0.0305**
			Time × Sex	F (5.143, 128.6) = 1.783	0.1188
Threat Conditioning Retrieval	Male	Two-way repeated measures ANOVA	Time	F (1,24) = 0.1791	0.6759
			**Treatment**	**F (1,24) = 610.4**	**< 0.0001**
			Time × Treatment	F (1, 24) = 9.663e-006	0.9975
	Female		Time	F (1,27) = 1.425	0.2430
			**Treatment**	**F (1,27) = 485.1**	**< 0.0001**
			Time × Treatment	F (1,27) = 0.5882	0.4498
	Non-stressed		Time	F (1, 26) = 0.03632	0.8503

			Sex	F (1, 26) = 0.7143	0.4057
			Time × Sex	F (1, 26) = 1.048	0.3154
	Stressed		Time	F (1, 25) = 0.1595	0.6930
			Sex	F (1, 25) = 0.6372	0.4322
			Time × Sex	F (1, 25) = 0.5813	0.4529
Extinction Retrieval	Male	Two-way repeated measures ANOVA	Time	F (1,24) = 0.7666	0.3899
			**Treatment**	**F (1,24) = 4.938**	**0.0360**
			Time × Treatment	F (1,24) = 0.06418	0.8022
	Female		Time	F (1,27) = 0.6941	0.4121
			**Treatment**	**F (1,27) = 8.581**	**0.0068**
			Time × Treatment	F (1,27) = 0.003325	0.9544
	Non-stressed		**Time**	**F (1, 26) = 5.619**	**0.0255**
			Sex	F (1, 26) = 0.1385	0.7128
			Time × Sex	F (1, 26) = 0.0001286	0.9910
	Stressed		Time	F (1, 25) = 0.4926	0.4893
			Sex	F (1, 25) = 1.412	0.2460
			Time × Sex	F (1, 25) = 0.06232	0.8049
Locomotor Activity (First Hour)	Male	Two-way repeated measures ANOVA	**Time**	**F (4.124, 49.49) = 47.87**	**<0.0001**
			Treatment	F (1, 12) = 0.03988	0.8451
			Time × Treatment	F (4.124, 49.49) = 0.5765	0.6860
	Female		**Time**	**F (6.463, 174.5) = 136.8**	**<0.0001**
			Treatment	F (1, 27) = 0.04163	0.8399
			Time × Treatment	F (6.463, 174.5) = 0.9346	0.4762
	Non-stressed		**Time**	**F (6.221, 124.4) = 96.33**	**<0.0001**
			Sex	F (1, 20) = 3.356	0.0819
			Time × Sex	F (6.221, 124.4) = 0.7684	0.6005
	Stressed		**Time**	**F (5.616, 106.7) = 66.03**	**<0.0001**
			Sex	F (1, 19) = 2.732	0.1148
			Time × Sex	F (5.616, 106.7) = 0.9809	0.4387
Locomotor Activity (Second Hour)	Male	Two-way repeated measures ANOVA	**Time**	**F (2.538, 30.45) = 11.78**	**<0.0001**
			Treatment	F (1, 12) = 2.261	0.1586
			Time × Treatment	F (2.538, 30.45) = 0.8750	0.4495
	Female		**Time**	**F (3.735, 100.9) = 22.24**	**<0.0001**
			Treatment	F (1, 27) = 3.328	0.0792
			Time × Treatment	F (3.735, 100.9) = 1.235	0.3010
	Non-stressed		**Time**	**F (2.521, 50.41) = 16.66**	**<0.0001**
			Sex	F (1, 20) = 3.963	0.0603
			Time × Sex	F (2.521, 50.41) = 1.532	0.2222
	Stressed		**Time**	**F (5.039, 95.75) = 16.26**	**<0.0001**
			Sex	F (1, 19) = 1.765	0.1997
			Time × Sex	F (5.039, 95.75) = 0.3698	0.8694
Cocaine-induced Hyperlocomotion	Male	Two-way ANOVA	**Time**	**F (1, 24) = 25.19**	**<0.0001**
			Treatment	F (1, 24) = 0.5997	0.4463
			Time × Treatment	F (1, 24) = 0.1822	0.6733
	Female		**Time**	**F (1, 54) = 65.15**	**<0.0001**
			Treatment	F (1, 54) = 1.692	0.1989
			Time × Treatment	F (1, 54) = 2.752	0.1029
	Non-stressed		**Time**	**F (1, 40) = 34.80**	**<0.0001**
			Sex	F (1, 40) = 2.289	0.1382
			Time × Sex	F (1, 40) = 0.1440	0.7064
	Stressed		**Time**	**F (1, 38) = 54.23**	**<0.0001**
			Sex	F (1, 38) = 0.1258	0.7248
			Time × Sex	F (1, 38) = 2.756	0.1051
Anxiety-like Behavior (First Hour)	Male	T-test	Treatment	t (12) = 0.7906	0.4445
	Female		Treatment	t (27) = 0.1376	0.8915
	Non-stressed		Sex	t (20) = 1.061	0.3014
	Stressed		**Sex**	**t (19) = 2.136**	**0.0459**
Anxiety-like Behavior (Second Hour)	Male	Unpaired T-test	Treatment	t (12) = 0.1026	0.9200
	Female	Mann–Whitney test	**Treatment**	**Mann–Whitney U = 57**	**0.0367**
	Non-stressed	Unpaired T-test	Sex	t (20) = 0.9702	0.3435
	Stressed	Unpaired T-test	Sex	t (19) = 2.039	0.0556

Table 3: Summary of Statistical Analysis: Sucrose Cohort

Behavioral Phase	Comparison	Analysis	Factor	F(df)	*p*-value
Self-Administration Active Lever Presses	Male	Two-way repeated measures ANOVA	**Time**	**F (4.606, 96.73) = 8.230**	**<0.0001**
			Treatment	F (1, 21) = 0.3896	0.5393
			Time × Treatment	F (4.606, 96.73) = 0.5656	0.7122
	Female		Time	F (4.478, 120.9) = 2.259	0.0595
			**Treatment**	**F (1, 27) = 5.220**	**0.0304**
			Time × Treatment	F (4.478, 120.9) = 0.4020	0.8274
	Non-stressed		**Time**	**F (3.869, 88.98) = 4.814**	**0.0016**
			**Sex**	**F (1, 23) = 26.37**	**<0.0001**
			**Time × Sex**	**F (3.869, 88.98) = 4.202**	**0.0040**
	Stressed		**Time**	**F (5.560, 139.0) = 5.341**	**<0.0001**
			**Sex**	**F (1, 25) = 27.41**	**<0.0001**
			**Time × Sex**	**F (5.560, 139.0) = 4.946**	**0.0002**
Self-Administration Inactive Lever Presses	Male	Two-way repeated measures ANOVA	**Time**	**F (3.747, 78.69) = 5.901**	**0.0004**
			Treatment	F (1, 21) = 3.551	0.0734
			Time × Treatment	F (3.747, 78.69) = 0.4633	0.7504
	Female		**Time**	**F (4.338, 117.1) = 4.962**	**0.0007**
			**Treatment**	**F (1, 27) = 10.18**	**0.0036**
			Time × Treatment	F (4.338, 117.1) = 1.084	0.3699
	Non-stressed		**Time**	**F (3.828, 88.05) = 5.175**	**0.0010**
			Sex	F (1, 23) = 0.05596	0.8151
			Time × Sex	F (3.828, 88.05) = 1.985	0.1067
	Stressed		**Time**	**F (4.438, 110.9) = 4.972**	**0.0007**
			Sex	F (1, 25) = 2.178	0.1525
			Time × Sex	F (4.438, 110.9) = 0.4496	0.7915
Extinction Active Lever Presses	Male	Two-way repeated measures ANOVA	**Time**	**F (2.323, 48.79) = 58.16**	**<0.0001**
			Treatment	F (1, 21) = 1.115	0.3029
			Time × Treatment	F (2.323, 48.79) = 0.8061	0.4689
	Female		**Time**	**F (2.403, 64.87) = 33.97**	**<0.0001**
			Treatment	F (1, 27) = 0.1743	0.6797
			Time × Treatment	F (2.403, 64.87) = 0.3453	0.7478
	Non-stressed		**Time**	**F (2.386, 54.88) = 36.23**	**<0.0001**
			Sex	F (1, 23) = 0.2330	0.6339
			Time × Sex	F (2.386, 54.88) = 1.071	0.3588
	Stressed		**Time**	**F (3.074, 76.84) = 55.96**	**<0.0001**
			Sex	F (1, 25) = 1.738	0.1994
			**Time × Sex**	**F (3.074, 76.84) = 3.649**	**0.0155**
Extinction Inactive Lever Presses	Male	Two-way repeated measures ANOVA	**Time**	**F (3.595, 75.50) = 5.615**	**0.0008**
			Treatment	F (1, 21) = 2.168	0.1557
			Time × Treatment	F (3.595, 75.50) = 1.435	0.2344
	Female		Time	F (2.408, 65.01) = 2.128	0.1178
			Treatment	F (1, 27) = 0.1411	0.7101
			Time × Treatment	F (2.408, 65.01) = 1.330	0.2729
	Non-stressed		Time	F (1.239, 28.51) = 1.510	0.2342
			Sex	F (1, 23) = 0.9987	0.3280
			Time × Sex	F (1.239, 28.51) = 0.8524	0.3870
	Stressed		**Time**	**F (4.975, 124.4) = 3.741**	**0.0035**
			Sex	F (1, 25) = 2.886	0.1017
			Time × Sex	F (4.975, 124.4) = 1.063	0.3841
Self-Administration Sucrose Pellets	Male	Two-way repeated measures ANOVA	**Time**	**F (4.096, 86.02) = 7.981**	**< 0.0001**
			Treatment	F (1, 21) = 0.002168	0.9633
			Time × Treatment	F (4.096, 86.02) = 1.268	0.2887
	Female		Time	F (4.754, 128.4) = 2.082	0.0752
			**Treatment**	**F (1, 27) = 9.783**	**0.0042**
			Time × Treatment	F (4.754, 128.4) = 0.7302	0.5954
	Non-stressed		Time	F (3.652, 83.98) = 2.417	0.0603
			**Sex**	**F (1, 23) = 41.76**	**< 0.0001**
			Time × Sex	F (3.652, 83.98) = 2.246	0.0767

	Stressed		**Time**	**F (4.692, 117.3) = 7.824**	**< 0.0001**
			**Sex**	**F (1, 25) = 27.51**	**< 0.0001**
			**Time × Sex**	**F (4.692, 117.3) = 5.343**	**0.0003**
Self-Administration Bodyweight %	Male	Two-way repeated measures ANOVA	**Time**	**F (2.063, 43.33) = 357.5**	**< 0.0001**
			Treatment	F (1, 21) = 0.04808	0.8285
			Time × Treatment	F (2.063, 43.33) = 0.4009	0.6785
	Female		**Time**	**F (5.589, 150.9) = 268.3**	**< 0.0001**
			Treatment	F (1, 27) = 0.4958	0.4874
			Time × Treatment	F (5.589, 150.9) = 1.155	0.3338
	Non-stressed		**Time**	**F (4.468, 102.8) = 373.0**	**< 0.0001**
			**Sex**	**F (1, 23) = 35.71**	**< 0.0001**
			**Time × Sex**	**F (4.468, 102.8) = 12.18**	**< 0.0001**
	Stressed		**Time**	**F (2.801, 70.02) = 285.6**	**< 0.0001**
			**Sex**	**F (1, 25) = 11.46**	**0.0023**
			**Time × Sex**	**F (2.801, 70.02) = 9.820**	**< 0.0001**
Cue-Primed Reinstatement	Male	Two-way ANOVA	**Time**	**F (1, 42) = 48.22**	**< 0.0001**
			Treatment	F (1, 42) = 3.057	0.0877
			Interaction	F (1, 42) = 3.375	0.0733
	Female		**Time**	**F (1, 54) = 74.21**	**< 0.0001**
			Treatment	F (1, 54) = 1.155	0.2873
			Interaction	F (1, 54) = 0.1247	0.7253
	Non-stressed		**Time**	**F (1, 46) = 50.83**	**< 0.0001**
			Sex	F (1, 46) = 1.585	0.2144
			Interaction	F (1, 46) = 1.017	0.3184
	Stressed		**Time**	**F (1, 50) = 65.31**	**< 0.0001**
			**Sex**	**F (1, 50) = 6.475**	**0.0141**
			**Interaction**	**F (1, 50) = 8.283**	**0.0059**
Threat Extinction	Male	Two-way repeated measures ANOVA	**Time**	**F (5.378, 112.9) = 47.69**	**< 0.0001**
			**Treatment**	**F (1, 21) = 93.37**	**< 0.0001**
			**Time × Treatment**	**F (5.378, 112.9) = 52.49**	**< 0.0001**
	Female		**Time**	**F (4.539, 122.6) = 15.70**	**< 0.0001**
			**Treatment**	**F (1, 27) = 23.32**	**< 0.0001**
			**Time × Treatment**	**F (4.539, 122.6) = 15.30**	**< 0.0001**
	Non-stressed		Time	F (5.658, 130.1) = 1.256	0.2841
			Sex	F (1, 23) = 3.740	0.0655
			Time × Sex	F (5.658, 130.1) = 0.6232	0.7021
	Stressed		**Time**	**F (5.112, 127.8) = 43.71**	**< 0.0001**
			Sex	F (1, 25) = 4.165	0.0520
			Time × Sex	F (5.112, 127.8) = 1.666	0.1459
Threat Conditioning Retrieval	Male	Two-way repeated measures ANOVA	Time	F (1, 21) = 2.168	0.1557
			**Treatment**	**F (1, 21) = 401.0**	**< 0.0001**
			Time × Treatment	F (1, 21) = 2.604	0.1215
	Female		Time	F (1, 27) = 3.903e-005	0.9951
			**Treatment**	**F (1, 27) = 366.2**	**< 0.0001**
			Time × Treatment	F (1, 27) = 0.1126	0.7398
	Non-stressed		Time	F (1, 23) = 2.544	0.1244
			Sex	F (1, 23) = 4.108	0.0544
			Time × Sex	F (1, 23) = 0.1438	0.7080
	Stressed		Time	F (1, 25) = 2.204	0.1501
			**Sex**	**F (1, 25) = 4.351**	**0.0473**
			Time × Sex	F (1, 25) = 1.654	0.2102
Extinction Retrieval	Male	Two-way repeated measures ANOVA	Time	F (1, 21) = 0.5355	0.4724
			Treatment	F (1, 21) = 0.8477	0.3677
			Time × Treatment	F (1, 21) = 3.258	0.0854
	Female		Time	F (1, 27) = 0.003001	0.9567
			**Treatment**	**F (1, 27) = 5.987**	**0.0212**
			Time × Treatment	F (1, 27) = 0.4450	0.5104
	Non-stressed		**Time**	**F (1, 23) = 7.315**	**0.0126**
			Sex	F (1, 23) = 0.6454	0.4300
			Time × Sex	F (1, 23) = 0.03488	0.8535
	Stressed		Time	F (1, 25) = 0.1923	0.6648
			**Sex**	**F (1, 25) = 5.051**	**0.0337**
			Time × Sex	F (1, 25) = 0.05276	0.8202

Locomotor Activity	Male	Two-way repeated measures ANOVA	**Time**	**F (5.254, 110.3) = 68.68**	**< 0.0001**
			Treatment	F (1, 21) = 0.4359	0.5163
			Time × Treatment	F (5.254, 110.3) = 1.063	0.3864
	Female		**Time**	**F (5.822, 157.2) = 78.77**	**< 0.0001**
			Treatment	F (1, 27) = 0.001999	0.9647
			Time × Treatment	F (5.822, 157.2) = 1.417	0.2131
	Non-stressed		**Time**	**F (5.609, 129.0) = 68.57**	**< 0.0001**
			Sex	F (1, 23) = 1.773	0.1961
			Time × Sex	F (5.609, 129.0) = 1.567	0.1665
	Stressed		**Time**	**F (6.510, 162.7) = 77.83**	**< 0.0001**
			Sex	F (1, 25) = 0.5750	0.4554
			Time × Sex	F (6.510, 162.7) = 1.964	0.0679
Anxiety-like Behavior	Male	Unpaired t-test	Treatment	t (21) = 0.3950	0.6968
	Female	Unpaired t-test	Treatment	t (27) = 1.261	0.2180
	Non-stressed	Unpaired t-test	**Sex**	**t (23) = 2.828**	**0.0095**
	Stressed	Unpaired t-test	Sex	t (25) = 1.103	0.2804
